# Binding Modes of Thalidomide Derivatives in Cereblon–Neosubstrate Complexes Revealed by Molecular Dynamics and Free Energy Calculations

**DOI:** 10.1021/acsomega.6c06210

**Published:** 2026-07-13

**Authors:** Verónica Martín, Milorad Andjelkovic, Carmen Barrientos, Iker Leon, Iñaki Tuñón

**Affiliations:** † Departamento de Química Física y Química Inorgánica, 16782 Universidad de Valladolid, 47011 Valladolid, Spain; ‡ Departament de Quìmica Fisìca, Universitat de València, 46100 Burjassot, Spain; § Grupo de Espectroscopía Molecular (GEM), Edificio Quifima, Laboratorios de Espectroscopia y Bioespectroscopia, Unidad Asociada CSIC, Parque Científico UVa, Universidad de Valladolid, 47011 Valladolid, Spain

## Abstract

Immunomodulatory drugs such as thalidomide and its derivatives act as molecular glues by binding to the E3 ligase substrate receptor cereblon (CRBN) and promoting the selective recruitment and degradation of specific neosubstrates. Despite extensive structural and experimental characterization, the molecular origin of ligand-induced neosubstrate selectivity remains incompletely understood. Here, we present a comparative computational study of ternary CRBN–ligand–neosubstrate complexes involving two biologically relevant neosubstrates, IKZF1 and SALL4, and five thalidomide derivatives displaying distinct experimental selectivity profiles. Using molecular dynamics simulations and thermodynamic integration calculations, we analyze ligand binding poses, CRBN–ligand interactions, and protein–protein contacts within the ternary complexes. Our results show that all ligands bind CRBN through a conserved interaction network within the thalidomide-binding domain, while direct and persistent ligand–neosubstrate contacts are not observed for the most stable binding modes. Moreover, the CRBN–neosubstrate interaction patterns remain largely unchanged across ligands, and calculated relative binding free energies do not reproduce experimentally observed selectivity trends. These findings suggest that ligand-induced selectivity cannot be explained solely by static interaction patterns in the ternary complex and point to the importance of additional factors, such as CRBN conformational dynamics and kinetic effects, in controlling neosubstrate recruitment.

## Graphical abstract

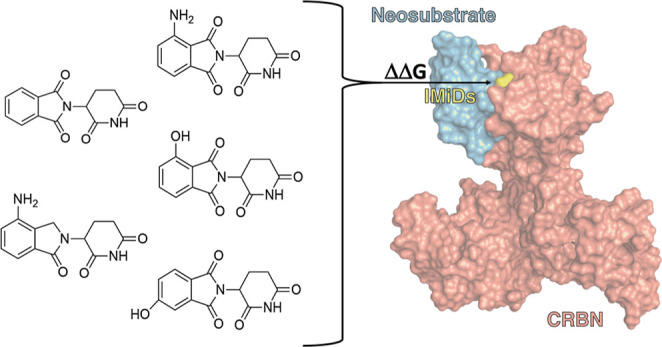

## Introduction

Thalidomide, lenalidomide, and pomalidomide are chiral immunomodulatory drugs (IMiDs) used in the treatment of multiple myeloma that exert their activity both by modulating the immune system and by acting directly on myeloma cells.^1–3^ The first known IMiD is thalidomide, which was introduced in the 1950s as a treatment for morning sickness in pregnant women and was vastly used. However, the newborns of these women developed a wide range of serious teratogenic effects, including limb defects, so the use of the drug was banned.^4,5^

Despite this tragedy, thalidomide was found effective for treating erythema nodosum, but it was not until 1990, when thalidomide’s potent antiangiogenic and immunomodulatory properties were identified and the drug was repurposed for the treatment of multiple myeloma.^3,6^ The mechanism of action of thalidomide remained uncertain until 2010, when Ito et al.^7^ identified the protein cereblon (CRBN) as the primary target of thalidomide and IMiDs in general. CRBN is a highly conserved protein across species consisting of 442 amino acids and three domains^8^ (see Figure 1a): the amino terminal domain or Lon domain (in green in Figure 1a), an intermedial helical bundle (in purple in Figure 1a), and a C-terminal thalidomide binding domain (TBD) (in red in Figure 1a) that has the pocket to insert thalidomide derivatives.^8,9^ CRBN is recruited to the Cullin-4-ROC1 ligase module by the adaptor protein DDB1, which subsequently leads to ubiquitination.^3,6,7,9^

**Figure 1:**
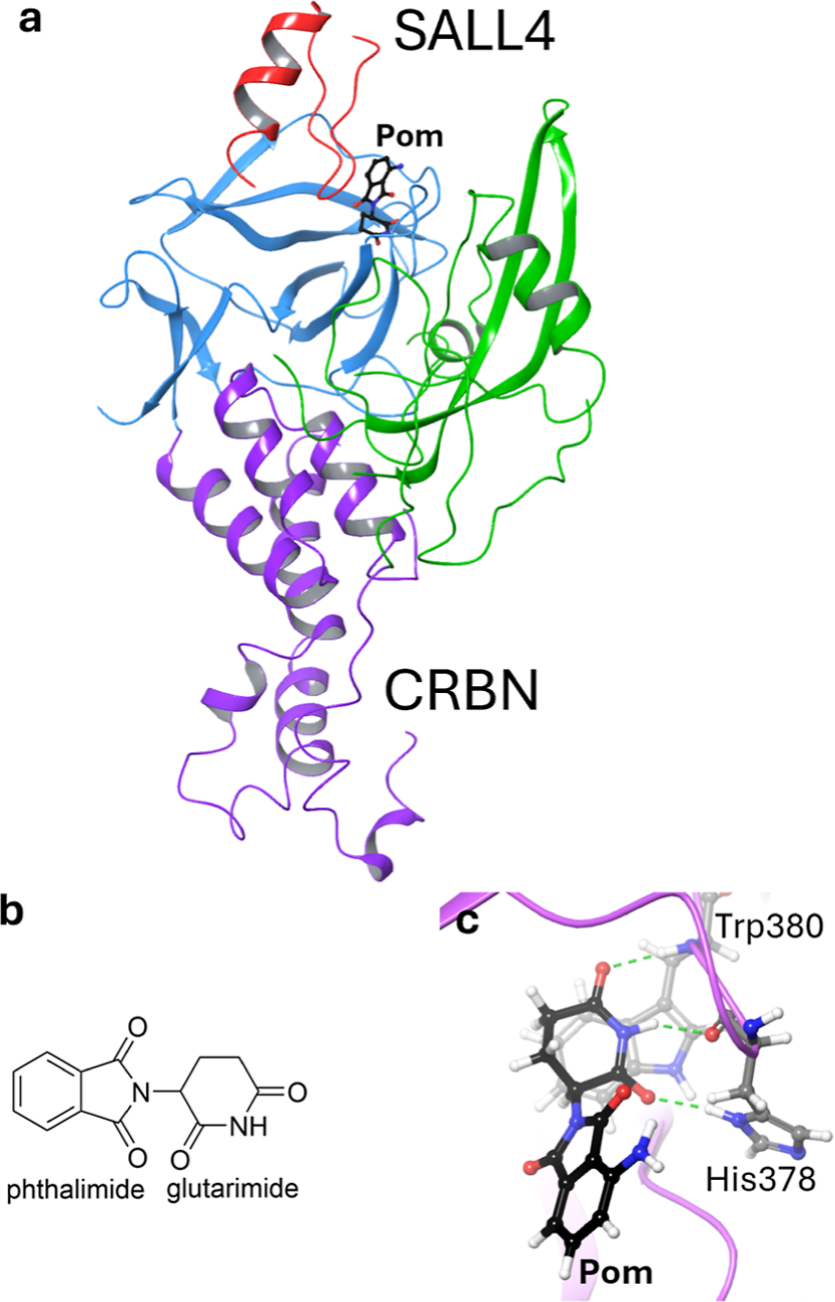
(a) Crystal structure 6UML with the proteins CRBN (with the Lon domain in green, an intermedial helical bundle in purple and the thalidomide binding domain in blue) and SALL4 (represented in red) and pomalidomide as a ligand. (b) Structure of thalidomide. (c) Hydrogen bonds between the ligand (pomalidomide) and active site residues His378 and Trp380.

Thalidomide and its derivatives present two structural elements: the glutarimide ring and the phthalimide ring (Figure 1b). All of them share the same glutarimide ring but differ in the phthalimide moiety, with various substituents, each one with a particular selectivity. The glutarimide ring commonly binds to CRBN in the TBD in an aromatic cage formed by three tryptophans, Trp380, Trp386, and Trp400. Inside this pocket, thalidomide forms two hydrogen bonds with the backbone of His378 and Trp380 and another hydrogen bond is formed with the side chain of His378 ^10,11^ (see Figure 1c). Only the glutarimide ring stays inside the pocket, while the phthalimide remains mostly outside.

One approach to developing new drugs is targeting specific harmful proteins to redirect them to the intrinsic protein degradation system present in our bodies, such as the ubiquitin E3 ligase system.^12^ IMiD molecules bind to the CRBN protein, which is a receptor of the E3 ligase, creating a favorable surface for other proteins or neosubstrates to bind for induced degradation. Recent studies have demonstrated that chemical modifications in the phthalimide ring can alter the selectivity and efficiency of IMiD-induced neosubstrate degradation, making the investigation of these interactions essential for novel drug design.^10,13–18^ Yamanaka et al.^16^ examined modifications on various positions of the phthalimide ring in lenalidomide and thalidomide derivatives. They employed an AlphaScreen-based interaction assay to evaluate the formation of the ternary complex involving CRBN, IMiD, and three different neosubstrates: IKZF1, SALL4, and PLZF. Neosubstrate IKZF1 encodes multiple isoforms of protein Ikaros consisting of six zinc finger structures that encompass two major protein domains that can bind to DNA.^19,20^ As a transcription factor, IKZF1 (Ikaros Family Zinc Finger 1) is involved in regulating the development and function of various immune cells and plays a crucial role in the immune system. Abnormal expression of IKZF1 may lead to different autoimmune diseases.^21,22^ In the presence of thalidomide and its derivatives, CRBN can selectively target Ikaros for ubiquitination and degradation. Similarly, SALL4 (Spalt-Like Transcription Factor 4) plays an essential role in maintaining the pluripotency and self-renewal properties of embryonic stem cells. After birth, its expression becomes downregulated and absent in most adult tissues. However, SALL4 is aberrantly re-expressed in numerous human diseases, with elevated levels observed in both hematological malignancies and solid tumors, including acute myeloid leukemia, breast cancer, and others.^23,24^ Structurally, SALL4 contains multiple zinc finger clusters, with isoforms SALL4A (1053 amino acids) and SALL4B (617 amino acids). In the presence of some thalidomide derivatives, CRBN can recruit this protein for its ubiquitination.^23,25^ Finally, PLZF (promyelocytic leukemia zinc finger) is another transcription factor with nine C_2_H_2_-type zinc finger domains, involved in a large range of developmental and biological processes, such as hematopoiesis, limb skeletal formation and immune regulation.^26,27^

Depending on the ligand bound to the CRBN protein, this protein can selectively target a different neosubstrate. Thalidomide, pomalidomide, and lenalidomide can recruit all the neosubstrates previously mentioned. However, the IMiD 5-hydroxythalidomide (see Figure 2) cannot recruit IKZF1 as a neosubstrate.^14^ Yamanaka et al.^16^ showed that thalidomide, lenalidomide, and pomalidomide establish stronger interactions with SALL4/PLZF than with IKZF1, while the complex formed with a lenalidomide derivative presenting a fluor group at 6-position interacts stronger with IKZF1 than with SALL4. Recent studies using crystallography and cryo-EM have shown that CRBN has two major conformations: an open state and a closed state. The open conformation has physiological relevance and is not just an artifact of crystallization.^8,9^ It has been hypothesized that ligand binding, especially at TBD, triggers an allosteric rearrangement that assists the transition from the open to the closed conformation, which is necessary for effective neosubstrate recruitment and degradation. Importantly, ligand binding alone is not always sufficient for closure; additional allosteric triggers and neosubstrate interactions may be required.^9^

**Figure 2:**
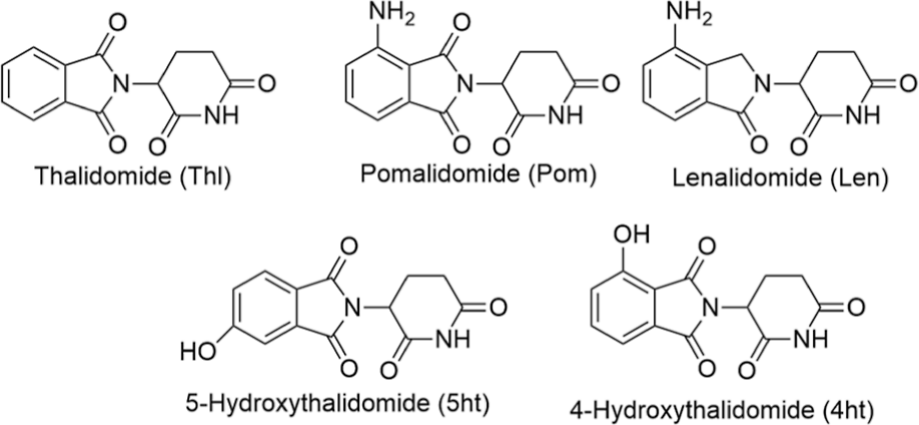
Chemical structures of thalidomide (Thl), pomalidomide (Pom), lenalidomide (Len), 5-hydroxythalidomide (5ht), and 4-hydroxythalidomide (4ht).

Computational simulations of the ternary CRBN–ligand–neosubstrate complex can be important to understand the origin of protein–protein selectivity and guide the design of new ligands targeting specific neosubstrates. However, these simulations are still challenging due to incomplete crystallographic structures of the ternary complex, the inherent flexibility of the involved proteins, and the size of the full systems (SALL4 has more than 1000 amino acids and IKZF1 has around 500 amino acids).^21,23^ Despite that, a computational simulation of lenalidomide forming a ternary complex with CRBN and neosubstrate CK1α concluded that the presence of this ligand increases the interactions between the two proteins due to a hydrophobic shielding of intermolecular hydrogen bonds.^28^ This observation can explain an increased activity but not the origin of selectivity. Other computational studies have shown that structure-based and free-energy approaches may capture part of the structure–activity relationships of CRBN molecular glues in favorable cases. Weiss et al.^29^ found that, for a congeneric series of cereblon E3 ligase modulators (CELMoDs) with similar binding modes, estimates of ternary complex stability provided useful information for classifying degradation activity. More recently, Dudas et al.^30^ formulated cooperativity in terms of binding free energies and used free energy perturbation calculations to identify highly active IKZF2 recruiters among functionalized pomalidomide derivatives. In both cases, the ligands contain extended chemical groups capable of engaging the CRBN–neosubstrate interface, so that changes in ternary complex stability can be directly connected, at least in part, to ligand-mediated interactions with the recruited protein.

In the present work, we explore whether this type of equilibrium free-energy analysis with more extensive sampling can be extended to chemically subtler modifications: a set of smaller thalidomide derivatives in which the structural differences are limited to local modifications on the phthalimide ring. By combining molecular dynamics simulations and thermodynamic integration, we aim to determine the preferred binding orientations of these ligands and to evaluate whether the resulting equilibrium interaction patterns are sufficient to explain the experimentally observed neosubstrate selectivity. We have carried out a systematic computational investigation of several CRBN–IMiD–neosubstrate ternary complexes, focusing on SALL4 and IKZF1 as neosubstrates. To pursue this goal, we selected two crystal structures as starting points for molecular dynamic calculations, PDB entries 6H0F^31^ and 6UML.^32^ The 6H0F structure includes CRBN with pomalidomide as a ligand and IKZF1 as a neosubstrate. Alternatively, the 6UML structure also presents CRBN and pomalidomide, but the neosubstrate is SALL4. To study the spectrum of ligand behavior, we have selected five thalidomide derivatives (see Figure 2). These include three drugs that are being used in clinical treatments: thalidomide (Thl), lenalidomide (Len), and pomalidomide (Pom). We also considered two additional hydroxyderivatives: 4ht, which in Yamanaka’s experiments^16^ does not exhibit activity with any of the two neosubstrates (SALL4 and IKZF1), and 5ht, which displays selective binding to SALL4 but not to IKZF1. This selection allows us to study the full range of behaviors observed experimentally: active ligands, inactive ligands, and selectively active ligands. Beyond the specific CRBN–IMiD–neosubstrate systems studied here, this work also addresses a methodological question of general relevance for the computational study of molecular glues: to what extent can equilibrium simulations of available ternary complex structures explain ligand-induced neosubstrate selectivity? In this context, alchemical free energy calculations are not only used to estimate relative binding affinities but also to discriminate between alternative ligand orientations in the thalidomide-binding domain. Several substituted thalidomide derivatives can adopt two plausible poses depending on the orientation of the phthalimide substituent, and these alternatives cannot be unambiguously ranked by inspection of static structures alone. By combining molecular dynamics simulations with thermodynamic integration, we aim to establish the most favorable ligand poses, rationalize their stabilization within CRBN, and assess whether these equilibrium binding modes and interaction patterns are sufficient to explain the experimentally observed selectivity profiles. In summary, this work aims to determine the preferred binding modes of representative thalidomide derivatives in CRBN–neosubstrate ternary complexes and to evaluate the extent to which these models can account for ligand-induced neosubstrate selectivity.

## Results and Discussion

To gain a deeper understanding of the mechanisms underlying the interactions between the ligands and the two proteins and ligand-induced selectivity observed in experimental studies, we prepared ten systems for simulations. We studied complexes CRBN-SALL4 and CRBN-IKZF1 with the five ligands presented in Figure 2. RMSD and RMSF values are presented in the SI (Figures S16–S25), supporting the structural stability of the simulated complexes during the production trajectories.

To facilitate the comparison between the simulations and the experimental ligand-induced selectivity profiles, Table 1 summarizes the neosubstrate recruitment behavior reported for the ligands considered in this work based on AlphaScreen (AS) recruitment assays.^16^ These experimental trends provide the reference framework against which the calculated ligand poses, interaction patterns, and relative binding free energies are discussed below. Except for thalidomide (Thl), which does not present substituents in the phthalimide ring, we simulated two different poses of all other ligands in the TBD: one pose with the amino or hydroxyl group oriented toward the neosubstrate (named here as ‘in’ orientation), and another with these groups oriented toward the CRBN protein (named as ‘out’). Figures S1 and S2 compare both orientations for pomalidomide. Therefore, a total of 18 complexes were simulated. To evaluate the relative binding energies of the ligands in different orientations, we performed alchemical transformations using Thermodynamic Integration (TI). These free energy calculations therefore serve two complementary purposes: first, to identify the thermodynamically preferred pose among plausible ligand orientations, and second, to compare the relative stabilization of different ligands in the CRBN–neosubstrate ternary complexes. The transformations were done starting from Thl, given that it presents a single binding pose because of the symmetry of the phthalimide ring, to each of the other ligands in the two different poses, both for CRBN-SALL4 and CRBN-IKZF1. Following this scheme, a total of 16 TI simulations were run with five replicas each (see Figure S3 and Tables S1 and S2 for details of TI simulations). Comparing the value of the ΔΔG_bind_ for the two different poses of each ligand, we have chosen the poses with the lowest binding free energy as the most favorable. We will here discuss in detail the favored poses, while the alternate, less favorable, poses are presented in the Figures S1, S2, S5–S8, S9, and S10 of SI. We first present the results for pomalidomide (Pom) because the comparison with the X-ray structures serves as a validation of the proposed protocol to identify the most favorable pose of the ligand.

**Table 1:** AlphaScreen-Based Biochemical Interaction Assay of CRBN–Neosubstrate Recruitment Induced by Thalidomide Derivatives, as Reported by Yamanaka et al;^16^ Values Are Averages from Three Independent Measurements

Ligand	relative AS signal	
	SALL4	IKZF1
thalidomide	48.2	19.4
pomalidomide	55.2	34.1
5-hydroxythalidomide	47.3	2.0
4-hydroxythalidomide	3.1	1.1
lenalidomide	37.4	31.8

### Pomalidomide

The relative binding free energy difference between the ‘out’ and ‘in’ poses is ΔΔ*G*_bind_ = −2.06 ± 0.12 kcal·mol^–1^ for SALL4 complex (see Table S1) and ΔΔ*G*_bind_ = −3.42 ± 0.04 kcal·mol^–1^ for IKZF1 (see Table S2). Then, TI calculations indicate that, between the two possible poses, the ‘out’ conformation is the most thermodynamically favorable, for both SALL4 and IKZF1 complexes. This finding agrees with the poses observed in the X-ray structures 6H0F^31^ and 6UML.^32^

The glutarimide ring of Pom forms stable hydrogen bonds with three CRBN residues: Asn351, His378, and Trp380 (Figure 3, panels c and d). The two carbonyl oxygen atoms of the glutarimide ring establish hydrogen bonds with residues Trp380 and His378. The interaction between the amino group of the glutarimide with the main chain carbonyl group of His378 is also conserved along the simulations, as well as between the carbonyl group of the phthalimide ring and Asn351. As indicated above, no stable hydrogen-bonding interactions are observed between the ligand and either of the two neosubstrates. This also agrees with X-ray structures 6H0F^31^ and 6UML,^32^ supporting the structural reliability of the ligand pose and the local interaction pattern. In the less favorable pose, Pom forms a hydrogen bond with the carbonyl group of one of the cysteines present in the zinc finger motif in both Cys412^S^ and Cys147^I^ (superindexes indicate residues belonging to SALL4 or IKZF1 neosubstrates) and minor interactions with other nearby residues of the neosubstrate chain; see Figures S1 and S2.

**Figure 3:**
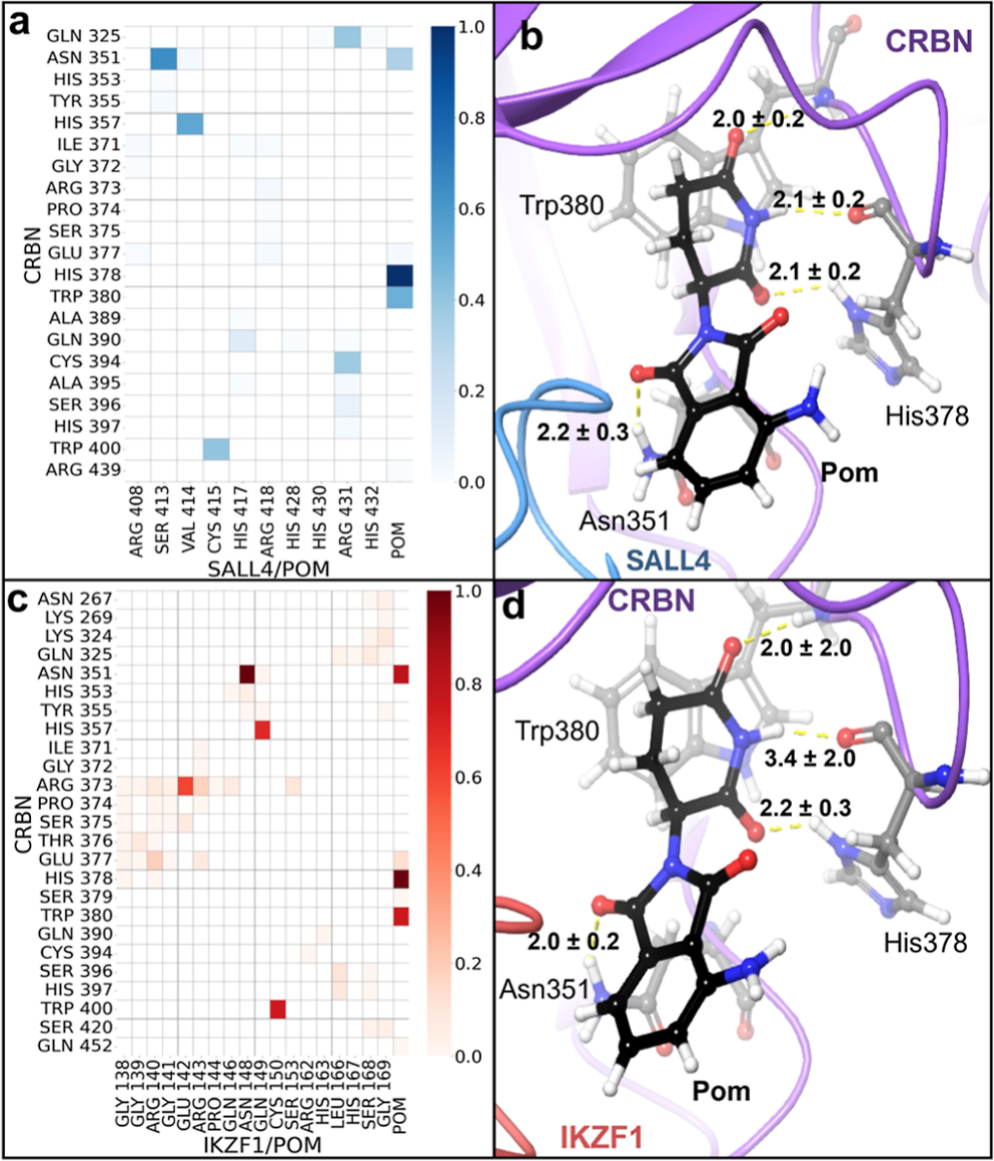
(a) Hydrogen bond heatmap between CRBN (*y*-axis), SALL4, and Pom (*x*-axis). The intensity of the color corresponds to the frequency of the contact. (b) Representative snapshot of Pom in complex with CRBN–SALL4, along with some average bond distances (in Angstroms). (c) Hydrogen bond heatmap between CRBN (*y*-axis), IKZF1, and Pom (*x*-axis). (d) Hydrogen bonds between Pom and CRBN, along with the average bond distances (in Angstroms) with IKZF1.

Regarding the interactions between CRBN and SALL4, the hydrogen bond interaction profiles (Figure 3a) reveal three key CRBN amino acids: Asn351, His357, and Trp400. In the complex with SALL4, Asn351 forms a hydrogen bond through its side-chain amino group with the main chain carbonyl group of Ser413^S^ (Figure S4a). The next prominent interaction between protein chains involves His357, whose imidazole ring forms a hydrogen bond with the backbone carbonyl group of Val414^S^ (Figure S4b). The last relevant interaction involves Trp400 (Figure 4c), specifically through the NH group present in the indole ring and the main chain carbonyl group of a cysteine residue present in the zinc finger (Cys415^S^). Previous studies^3,8,10,14^ remarked on the importance of Trp400 not only because of its ability to form hydrogen bonds with the neosubstrates but also for its role as one of the residues that form the TBD. In addition, Arg351^S^ interacts with two CRBN residues, Gln325, and Cys394 (which form part of the CRBN zinc finger).

**Figure 4:**
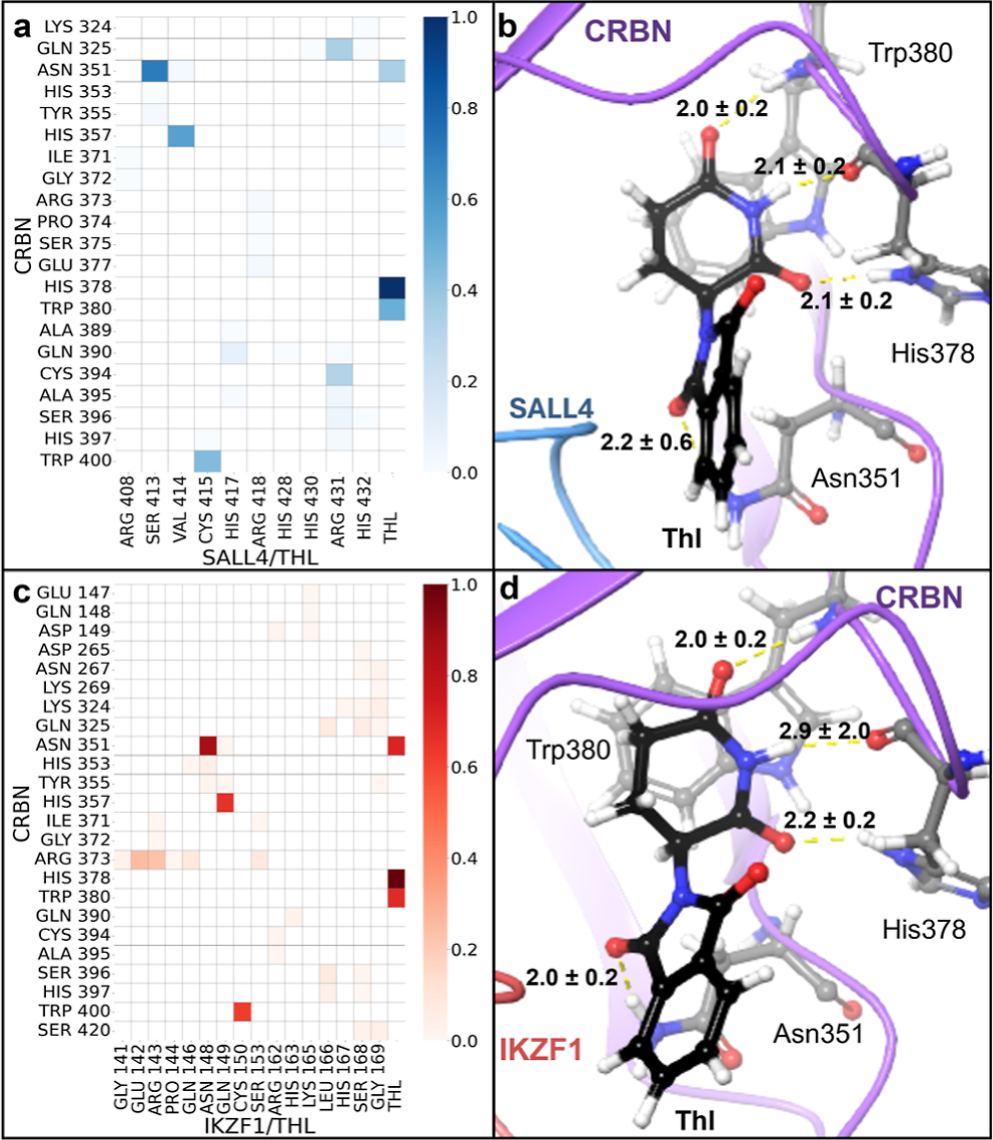
(a) Hydrogen bond heatmap between CRBN, SALL4, and Thl. (b) Representative snapshot of Thl in complex with CRBN-SALL4, along with some average bond distances (in Angstroms); (c) hydrogen bond heatmap between CRBN, IKZF1 and Thl. (d) Hydrogen bonds between Thl and CRBN, along with the average bond distances (in Angstroms) with IKZF1.

The CRBN–IKZF1 interface displays interaction patterns similar to those observed for SALL4 (see Figure 3c,d). Asn351 side chain amino groups form a hydrogen bond with the mainchain carbonyl group of Asn148^I^ (Figure S4d), while His357 interacts via its imidazole ring with the mainchain carbonyl group of Gln149^I^ (Figure S4e). Trp400 again interacts with the carbonyl group of Cy150^S^ that belongs to the zinc finger of the protein IKZF1 (Figure S4f). It should be noted that, in this case, Arg373 establishes a salt-bridge interaction with the carboxyl group of Glu142^I^. Arg373 also establishes minor interactions (see Figure 3c) with a range of residues of IKZF1 (from Gly138^I^ to Gln148^I^), which contributes to the stability of the complex.

Overall, our results indicate that pomalidomide binding is primarily mediated by conserved residues in CRBN, with secondary structural elements (such as the zinc finger) contributing to the stabilization of the complex, consistent with the crystallographic data. However, we did not observe direct interactions between the ligand and either of the two neosubstrates that could explain any ligand-induced selectivity.

### Thalidomide

In the case of thalidomide (Thl), only one binding pose has been explored because the phthalimide ring is symmetric with respect to its rotation around the bond connecting with the glutarimide ring. Thl presents almost the same interaction pattern with CRBN compared to Pom. The ligand binds to CRBN with no noticeable contacts with the neosubstrates (see Figure 4a,4c). As in the preceding case, Trp380 and His378 are key residues establishing hydrogen bond interactions with the glutarimide ring of thalidomide, while Asn351 forms a hydrogen bond with one of the phthalimide ring’s carbonyl groups (see Figure 4b,d for a representative pose in the complex with SALL4 and IKZF1, respectively).

The interface CRBN–SALL4 shows no significant differences in hydrogen bond patterns compared to the complex with Pom (see Figures 4a and 3a). However, there is a slight variation in the CRBN–IKZF1 interactions. Although CRBN still engages the same region of IKZF1, the hydrogen bond involving Arg373 with Glu142^I^ is less persistent when Thl is present, forming also contacts with the mainchain carbonyl group of Arg143^I^ during a significant fraction of the simulations (compare Figure 3c (Pom) and 4c (Thl)).

### 4-Hydroxythalidomide

As in the case of Pom, we considered two possible orientations of the phthalimide ring of 4-hydroxythalidomide (4ht) in the active site. The ‘out’ pose presented in Figure 5 corresponds to the pose with the most favorable binding free energy (ΔΔG_bind_ = −1.21 ± 0.08 kcal·mol^–1^ for SALL4 (Table S1) and ΔΔG_bind_ = −1.92 ± 0.09 kcal·mol^–1^ for IKZF1 (Table S2)). A comparison between both poses (‘in’ and ‘out’) is shown in Figures S5 and S6, the most prominent difference being the interactions with the neosubstrates. While the ‘out’ pose do not interact with the neosubstrates, the unfavorable ‘in’ pose forms a hydrogen bond between the hydroxyl group of the phthalimide ring and the mainchain carbonyl groups of the cysteines of each neosubstrate: Cys412^S^ in SALL4 and Cys 147^I^ in IKZF1. For the ‘out’ pose, the same set of residues reported in previous cases also appear to be involved in the formation of hydrogen bond interactions between 4ht and CRBN: Trp380, His378, and Asn351 (see Figure 5a,c).

**Figure 5:**
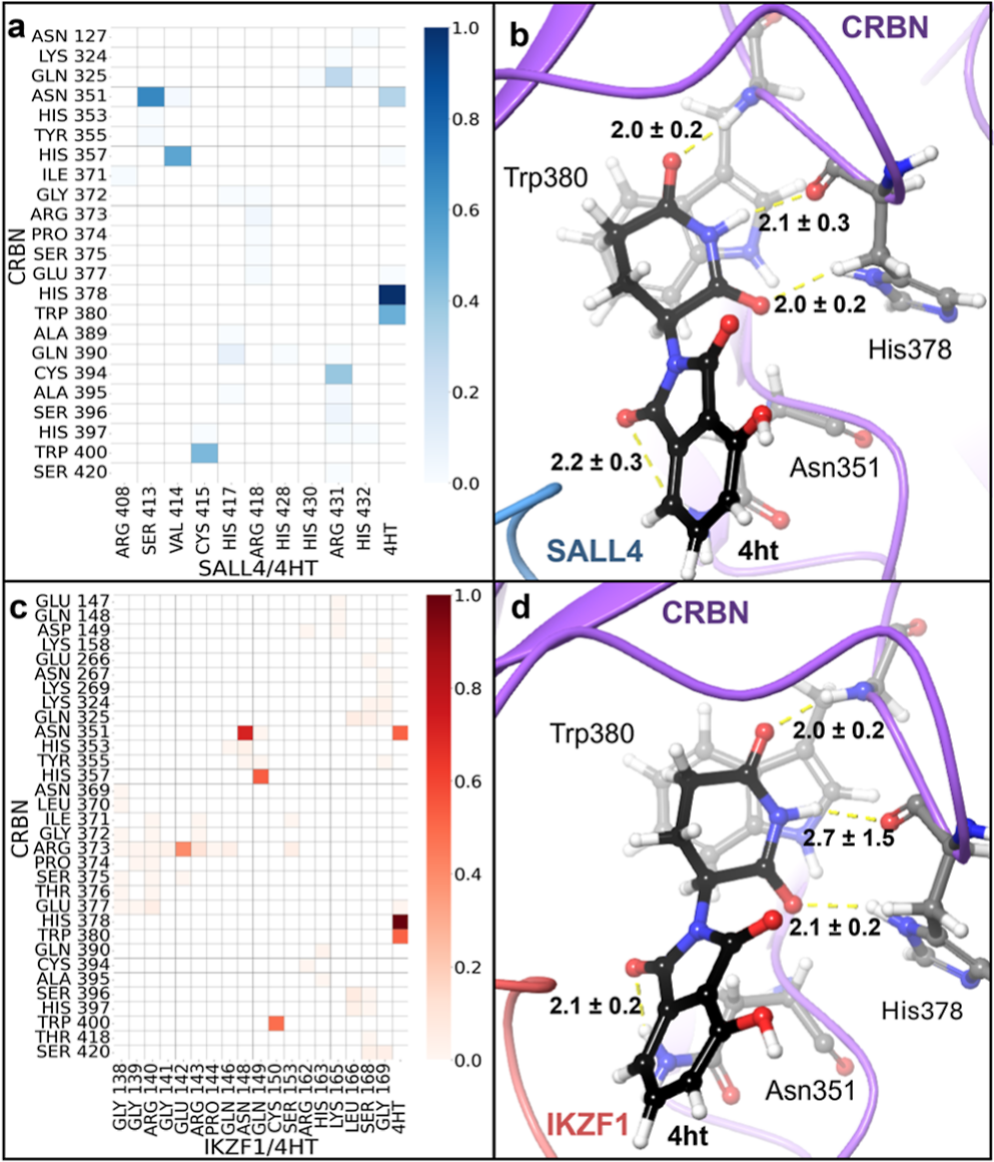
(a) Hydrogen bond heatmap between CRBN, SALL4, and 4ht. (b) Representative snapshot of 4ht in complex with CRBN–SALL4, along with some average bond distances (in Angstroms). (c) Hydrogen bond heatmap between CRBN, IKZF1, and 4ht. (d) Hydrogen bonds between 4HT and CRBN, along with the average bond distances (in Angstroms) with IKZF1.

Regarding the CRBN–SALL4 interactions, the hydrogen-bonding pattern is very similar to that reported in the previous cases. However, in comparison with Pom, the interaction between Arg431^S^ and Gln325 is slightly less pronounced (Figure 3a) when 4ht is the ligand. Subtle differences also emerge for the CRBN–IKZF1 interactions in the ternary complex with 4ht. The same set of residues are involved in hydrogen bonds between both proteins; however, the intensity of these interactions is reduced when 4ht is present, particularly between Arg373 and Glu142^I^ (compare Figures 5c (4ht) and 3c (Pom)).

### 5-Hydroxythalidomide

We also simulated two possible orientations of 5-hydroxythalidomide (5ht) in the active site: the ‘out’ pose, where the 5-hydroxyl substituent is oriented toward the CRBN chain, and the ‘in’ pose, with the 5-hydroxyl substituent oriented toward the neosubstrate. The free energy differences between both poses are ΔΔ*G*_bind_ = −1.28 ± 0.08 kcal·mol^–1^ in the complex with SALL4 (Table S1) and ΔΔ*G*_bind_ = −0.23 ± 0.02 kcal·mol^–1^ for IKZF1 (Table S2), being always the ‘out’ pose the most favorable. Both poses are shown in Figures S7 and S8, while the most stable one is also presented in Figure 6b,d. Notably, the predicted most stable pose is consistent with the solved X-ray structure (PDB ID 7BQV^14^), displaying an excellent overlap between them (see Figure S13).

**Figure 6:**
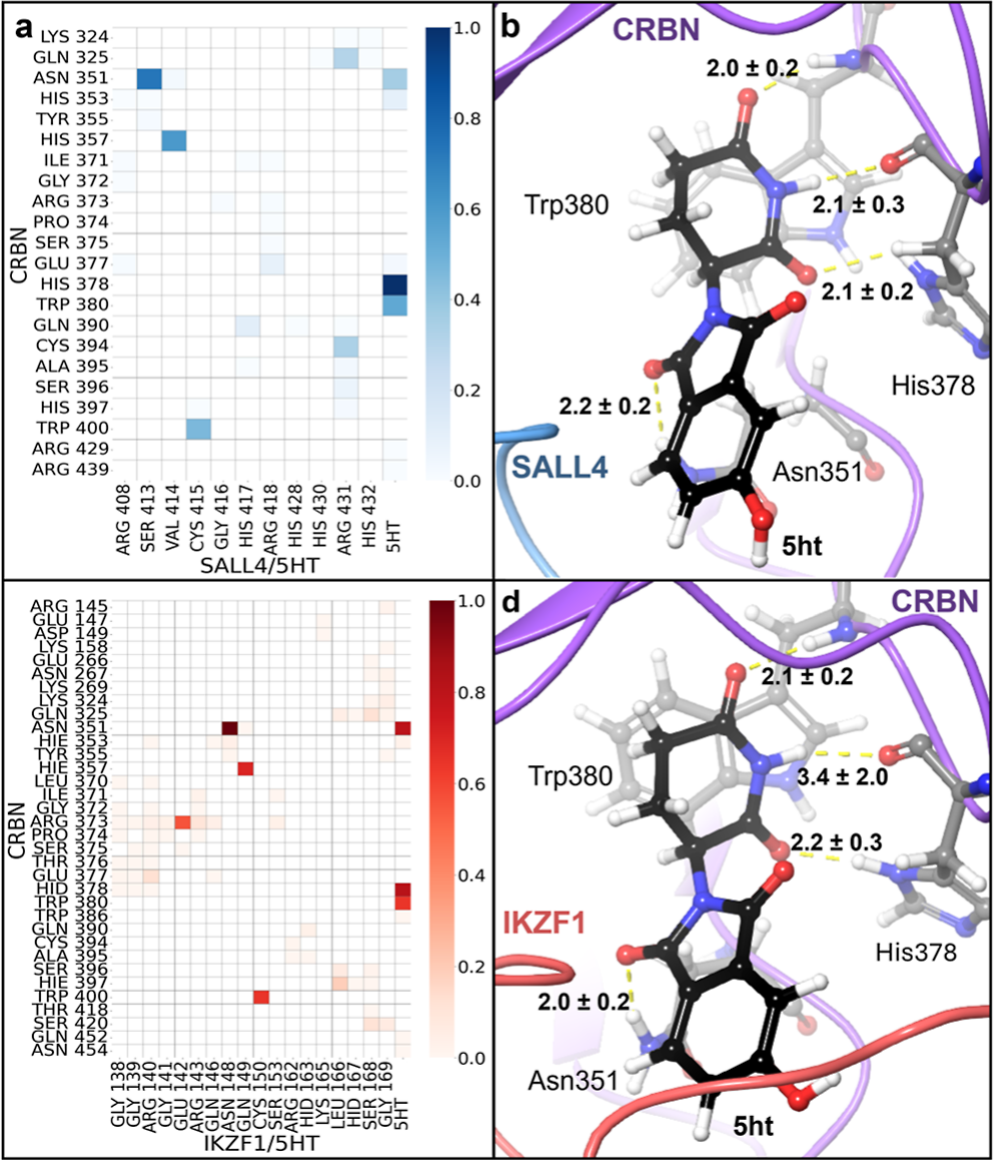
(a) Hydrogen bond heatmap between CRBN, SALL4, and 5HT. (b) Representative snapshot of 5ht in complex with CRBN–SALL4, along with some average bond distances (in Angstroms). (c) Hydrogen bond heatmap between CRBN, IKZF1, and 5ht. (d) Hydrogen bonds between 5HT and CRBN, along with the average bond distances (in Angstroms) with IKZF1.

Figure 6a,c shows that the interactions between the ligand and CRBN remain the same as observed in previous cases for both complexes with the two neosubstrates. The interactions with CRBN residues His378, Trp380, and Asn351, previously identified as the most relevant, are maintained in both complexes, regardless of the neosubstrate.

Regarding CRBN–neosubstrate interactions, no significant differences are observed in the hydrogen bonds established between SALL4 and CRBN when 5ht is present with respect to the other ligands. The 5ht complex with the CRBN–IKZF1 complex also exhibits a comparable hydrogen bond network, with conserved strong interactions with CRBN residues Asn351 and His357. In this last case, we observe a slight reduction in the frequency of contacts with His378 and increased with Cys394, indicating minor ligand-dependent differences at the CRBN–IKZF1 interface (see Figure 6c).

### Lenalidomide

Lenalidomide (Len) exhibits larger differences than the other ligands when compared to Pom, probably because this ligand lacks one of the carbonyl groups of the phthalimide ring. Furthermore, notable differences are observed in the binding free energy calculations between the two possible poses, with one of them being much more favorable than the other. The ‘out’ pose is significantly more stable than the ‘in’ pose, ΔΔ*G*_bind_ = −7.45 ± 0.44 kcal·mol^–1^ for SALL4 (Table S1) and ΔΔ*G*_bind_ = −8.08 ± 0.34 kcal·mol^–1^ for IKZF1 (Table S2). Both poses are depicted in Figures S11 and S12, while the most stable pose is additionally presented in Figure 7. This most favorable pose coincides with that observed by Miñarro et al.^28^ and the crystallographic data of the complex between CRBN and lenalidomide.

**Figure 7:**
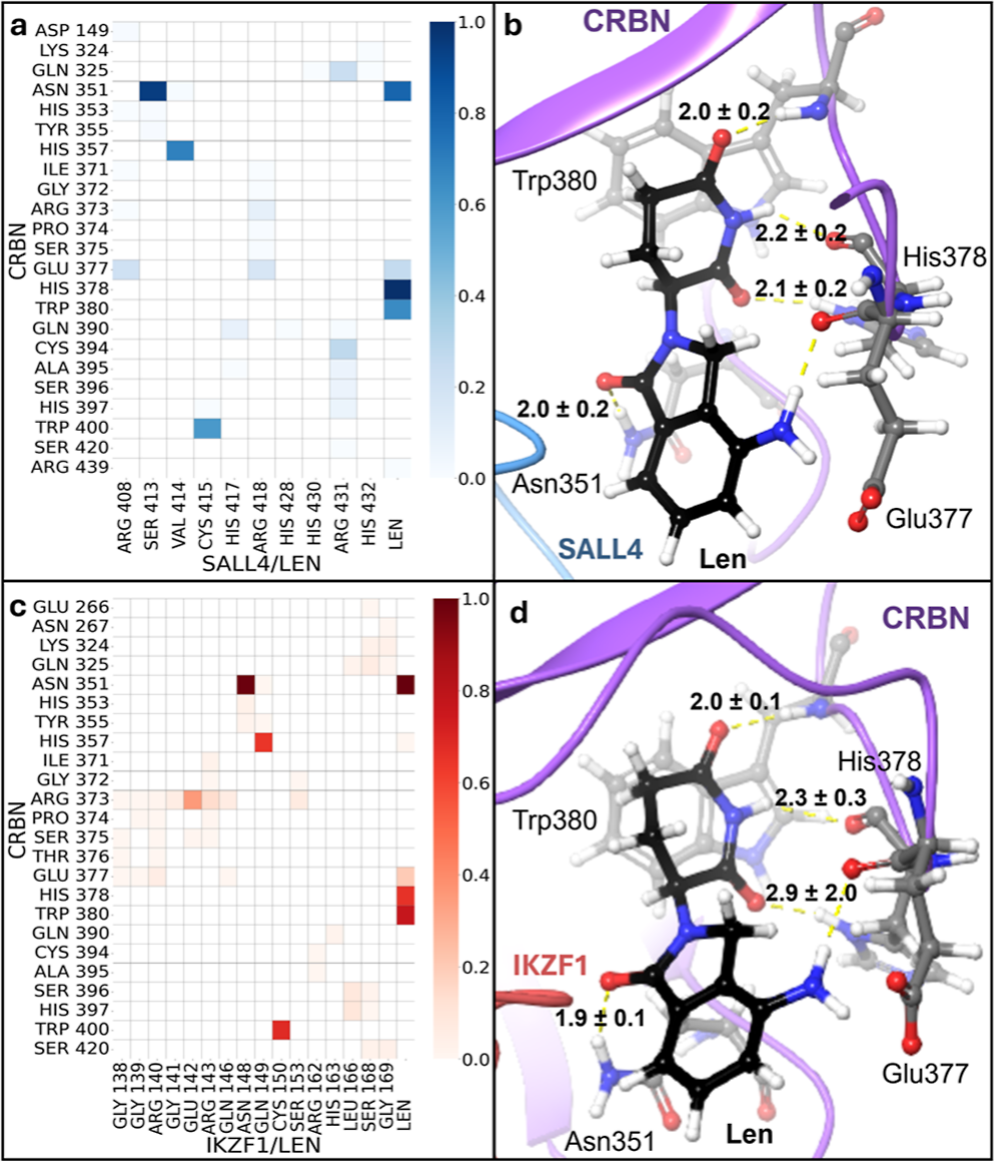
(a) Hydrogen bond heatmap between CRBN, SALL4, and Len. (b) Representative snapshot of Len in complex with CRBN–SALL4, along with some average bond distances (in Angstroms). (c) Hydrogen bond heatmap between CRBN, IKZF1, and Len. (d) Hydrogen bonds between Len and CRBN, along with the average bond distances (in Angstroms) with IKZF1.

In the case of Len, the ‘out’ pose presents the amino group of the phthalimide ring oriented toward CRBN, forming a permanent hydrogen bond between this amino group and the mainchain carbonyl group of Glu377. This contrast to the case of Pom where this group establishes a hydrogen bond with a water molecule present in the active site that prevents the formation of a direct interaction with the amine group of the ligand. The presence of this water molecule in the case of Pom has been corroborated calculating the radial distribution functions around the carbonyl group of Glu377 when Pom is present in the active site (see Figure S9a for SALL4 and Figure S10a for IKZF1). This water molecule is absent in the case of Len (see Figure S9b for SALL4 and Figure S10b for IKZF1), allowing the formation of a direct ligand–CRBN contact, in agreement with the observations obtained in a previous study.^28^ Meanwhile, the only carbonyl group present in the phthalimide ring also forms a permanent hydrogen bond contact with the Asn351 side chain.

As in the preceding cases, only minor variations are observed in protein–protein interactions within the ternary complexes. The analysis of protein–protein hydrogen bond interactions for the CRBN–Len–SALL4 complex (compared with that for Pom) reveals small differences. More intense interactions are observed between key residues pairs (Asn351–Ser413^S^, His357–Val414^S^, and Cys415^S^–Trp400), while lower intensity is observed for interactions between Arg431^S^ of SALL4 and residues Gln325 and Cys394 of CRBN. In the CRBN–IKZF1 complex with Len, we can appreciate that the main hydrogen bonds interactions, Asn351–Asn148^I^, His357–Gln149^I^, and Trp400–Cys^I^150, are similar to those observed with Pom. However, interactions between Arg373 and neosubstrate’s residues appear to be weaker in the CRBN–IKZF1 complex with Len than with Pom.

## Discussion

An important outcome of the present calculations is the assignment of the preferred binding orientations of the substituted thalidomide derivatives within the CRBN thalidomide-binding domain. For Pom, 4ht, 5ht, and Len, two plausible orientations can be generated by rotating the substituted phthalimide moiety, leading either to an “in” pose, with the substituent oriented toward the neosubstrate or to an “out” pose, with the substituent oriented toward CRBN. While both orientations are structurally plausible, the TI calculations consistently identify the “out” orientation as the most stable one for the ligands and neosubstrate complexes studied here. This result provides a quantitative basis for pose assignment and shows that the preferred binding modes are dominated by ligand–CRBN interactions rather than by direct ligand–neosubstrate contacts. Summarizing the results of the different CRBN–ligand–neosubstrate complexes presented above, we can conclude that three CRBN residues (Trp380, His378, and Asn351) critically contribute to ligand–protein binding. This observation is in agreement with the data available from X-ray structures^31,32^ and previous computational analysis.^28^ Slight differences are observed from the analysis of ligand–CRBN interactions: Len establishes also direct hydrogen bond interactions with mainchain Glu377 carbonyl group that are not observed in other ligands. In the most stable poses, none of the studied ligands display noticeable direct interactions with the neosubstrate, suggesting that direct ligand–neosubstrate contacts are unlikely to be responsible for the observed ligand-induced selective activity of CRBN with different neosubstrates.

In addition, no significant differences were observed in the direct interactions between CRBN and the neosubstrates. The most relevant contacts in all cases are those involving residues Asn351, His357, and Trp400 of CRBN with residues Ser413^S^, Val414^S^, and Cys415^S^, in the case of SALL4 and Asn148^I^, Glu149^I^, and Cys150^I^, in the case of IKZF1. In this last case, all ternary CRBN–ligand–IKZF1 complexes, except in the case of Thl, also present a contact between Arg373 and Glu142^I^. Therefore, ligand-induced CRBN–neosubstrate interactions also seem insufficient to explain the ligand-induced selectivity experimentally observed in CRBN activity.^14,16^

In order to try to go beyond the qualitative description obtained from the analysis of hydrogen bond contacts, we computed the free energy changes for the alchemical transformations of Thl into Pom, 4ht, 5ht, and Len. The observed changes in the binding free energies (Table 2) were useful to identify the most stable pose of each ligand and could also be useful to try to find correlations with the observed recruitment profiles between CRBN and neosubstrates.

**Table 2:** Thermodynamic Integration Results Corresponding to the Alchemical Transformation of Thalidomide into the Most Stable Pose of Different Ligands in the Ternary Complexes with CRBN and the Two Neosubstrates Studied Here^a^

TI transformation	ΔΔ*G*_bind_ (kcal/mol)	
	SALL4	IKZF1
Thl → Pom	–0.13 ± 0.03	–0.47 ± 0.02
Thl → 4ht	–0.01 ± 0.02	0.37 ± 0.05
Thl → 5ht	0.05 ± 0.04	–0.44 ± 0.01
Thl → Len	–2.32 ± 0.12	–3.96 ± 0.15

a The standard deviation corresponds to the average of five replicas (see Supporting Information).

According to Yamanaka et al.,^16^ variations in the phthalimide ring directly influence the CRBN activity toward different neosubstrates. Consistently, Pom shows slightly higher affinity than Thl for both neosubstrates, in agreement with the observed changes in the binding free energy in the transformation Thl →Pom (see Table 1) Also, in those experiments, 4ht was shown to substantially reduce CRBN affinity for both SALL4 and IKZF1 neosubstrates, when compared to Thl. Our free energy calculation for the Thl →4ht transformation are consistent with a diminished affinity only for the neosubstrate IKZF1, but no variation is observed for SALL4. Also, in these experiments, 5-hydroxy-thalidomide (5ht) was found to enable the recruitment of SALL4 but not IKZF1.^14,16^ However, our alchemical transformations show that the binding free energy of the ligands in the ternary complexes with SALL4 is almost identical for 4ht, 5ht, and Thl. In the case of IKZF1, our simulations even point to a stronger binding in the ternary complex when Thl is transformed to 5ht. Previous structural studies suggested that the change of Val411^S^ (SALL4) to Gln146^I^ (IKZF1) can produce the unbinding of 5ht.^14^ However, our free energy calculations show the opposite trend. Regarding Len, experiments show no significant differences with respect to Thl, while our simulations suggest a large increase in the binding free energy, even more pronounced in the case of IKZF1.

The calculated relative binding free energies presented in Table 2 do not reproduce the experimentally observed selectivity trends for the smaller thalidomide derivatives studied here. This result should be interpreted in light of recent computational studies showing that structure-based and free-energy approaches can capture part of the activity trends for more extended CRBN molecular glues. Weiss et al.^29^ reported that, for congeneric CELMoD series with similar binding modes, in silico estimates of ternary complex stability can provide useful information for classifying degradation activity. However, they also reported that pomalidomide-based analogues were outliers in their Free Energy Perturbation (FEP) analysis: although FEP predicted less favorable ternary binding than for the corresponding lenalidomide-based analogues, their cellular degradation potencies were similar. This discrepancy was attributed to the possibility that more favorable physicochemical properties, such as improved permeability, may partially compensate for weaker ternary binding. Similarly, Dudas et al.^30^ showed that FEP-based estimates of ternary binding and cooperativity can identify the most effective IKZF2 recruiters among functionalized pomalidomide derivatives. In both cases, however, the ligands contain larger substituents or charged groups that engage the CRBN–neosubstrate interface more directly, thereby providing stronger energetic differences for structure-based methods to detect. However, their results also indicate that, while FEP calculations successfully identified the strongest recruiters, more subtle activity differences within the series were not fully resolved, particularly among ligands with comparable profiles. Our TI protocol involved substantially longer sampling per alchemical window than the protocols reported in recent related studies. Therefore, the absence of a clear correlation with experimental selectivity is unlikely to be attributable simply to very short sampling times. Rather, it may reflect the more subtle nature of the chemical modifications considered here, the limited ligand–neosubstrate interaction surface of these smaller IMiDs, and/or contributions from mechanisms not captured by equilibrium ternary-complex binding free energies and discussed below. Even in studies reporting partial success of FEP-based approaches,^29,30^ subtle activity differences among closely related molecular glues remain difficult to capture reliably. This suggests that FEP-based approaches may be more effective at detecting large changes in ternary complex stabilization than subtle differences arising from small local modifications of the ligand scaffold.

Thus, the present replica-exchange alchemical transformations do not fully reproduce the experimental selectivity trends, suggesting that additional factors beyond our simulations may contribute to this selectivity. It must be considered that the structural models are incomplete, and that neosubstrates contain large intrinsically disordered regions that are not resolved in the crystallographic structures.^22,23^ It is therefore possible that the use of these incomplete models does not allow capturing the full effect of the ligand on CRBN selectivity toward the neosubstrates. However, the lack of correlation between the calculated relative binding free energies and the experimental selectivity profiles should not be merely interpreted as a possible failure of the calculations to reproduce activity data. The experimental comparison is based on AlphaScreen recruitment assays^16^ that may depend on possible differences in solubility or effective compound availability in the assay medium. The observed discrepancy can also provide a mechanistic constraint on the origin of ligand-induced selectivity in these systems. The simulations show that, once the most stable ligand poses are identified, neither direct ligand–neosubstrate contacts nor simple equilibrium stabilization of the ternary complexes is sufficient to account for the experimental trends. Thus, the determinants of selectivity could involve physical effects that are not captured by the present equilibrium models, such as conformational selection or induction in CRBN, the open–closed transition of the receptor,^9^ the contribution of unresolved regions of the neosubstrates (only the resolved regions, corresponding to the zinc finger fragments, were considered in our simulations), or kinetic effects associated with ligand and neosubstrate association and dissociation.^9^ These possibilities are not considered in the molecular dynamic simulations carried out in this study and may also contribute to the observed ligand-induced selectivity.

## Conclusions

In this work, we have performed a comparative computational study of ternary CRBN–ligand–neosubstrate complexes involving two biologically relevant neosubstrates, IKZF1 and SALL4, and a representative set of thalidomide derivatives spanning active, inactive, and selectively active ligands. Using molecular dynamics simulations combined with thermodynamic integration calculations, we characterized the best ligand binding poses, identified conserved CRBN–ligand interaction patterns, and analyzed how different IMiDs modulate the protein–protein interfaces within the ternary complexes. Our results show that ligand binding is consistently dominated by conserved interactions with CRBN residues in the thalidomide-binding domain (TBD). In the most favorable binding poses, however, we do not find evidence for stable or persistent hydrogen-bond interactions between the ligand and the neosubstrate that could provide a direct explanation for the experimentally observed selectivity. This distinction is important because occasional proximity or transient contacts at the CRBN–neosubstrate interface do not necessarily imply a sustained ligand-mediated interaction capable of controlling neosubstrate recruitment. In this regard, our predictions agree with available structural data. Moreover, the CRBN–neosubstrate interaction networks remain largely unchanged across ligands, suggesting that simple equilibrium interaction patterns within the ternary complex are insufficient to account for the experimentally observed ligand-induced selectivity.

Despite capturing stable ternary complexes and reproducing key structural features, the discrepancy between the calculated binding free energies and the experimentally observed ligand-induced selectivity opens new questions that will require further investigation beyond the present computational approach. Comparison with previous, partly successful free energy simulations suggests that, despite the extended sampling times used here, these approaches may be more effective at detecting large changes than subtle differences arising from small local modifications of the ligand scaffold. Another source of divergence may arise from the fact that the measured experimental signal should not be interpreted as a direct thermodynamic binding free energy of the ternary complex. In addition, unresolved regions of the neosubstrates, the kinetics of the binding/unbinding process, and the intrinsic flexibility of CRBN, in particular the possibility of open–closed conformational transitions, could play a critical role in the observed ligand-induced selectivity. These effects are not captured in the present simulations. Future work should therefore focus on incorporating larger and more complete CRBN models, explicitly sampling open–closed conformational equilibria, and exploring kinetic effects associated with ligand binding and neosubstrate recruitment. Such approaches will be essential to achieve a more comprehensive mechanistic understanding of ligand-induced selectivity and to guide the rational design of next-generation molecular glues.

## Methods

### Classical Molecular Dynamics (MD) Simulations

The ternary −CRBN–neosubstrate–ligand complexes were prepared using Maestro.^33^ Models for the CRBN, SALL4, and IKZF1 were built starting from the crystallographic structure PDB ID: 6H0F^31^ and 6UML^32^ from the Protein Data Bank, both containing Pomalidomide in the TBD.^34–36^ Standard protein preparation protocols were followed, including the removal of duplicate protein chains from PDB entry 6H0F,^31^ selection of the most complete CRBN–IKZF1 structure, and removal of crystallization buffer components and salt ions. Additionally, the DNA damage-binding protein 1 was removed in all systems. The terminal residues of CRBN were capped and missing residues of CRBN were built using PRIME.^37,38^ The crystallized structures of the neosubstrates correspond only to the second zinc finger (ZF2) among the multiple zinc fingers of SALL4 ^32^ and the ZF2 domain of the multi-zinc finger transcription factor of IKZF1.^31^ In our simulations, we used only the experimentally resolved regions of the neosubstrates available in the 6H0F and 6UML PDB structures. The protonation states of all residues were calculated with PROPKA3,^39,40^ and the protonation states of the residues in CRBN in all models was kept the same. The ff14SB^41^ and gaff^42^ force fields were used to assign atom types for the protein and the ligands, respectively. Partial charges for ligands were derived using the RESP^43,44^ protocol at the HF/6-311++G­(2df,2pd) level of theory, as calculated with Gaussian16.^45^ The Zn^2+^ cation bound to CRBN, IKZF1, and SALL4 was modeled using ZAFF.^46^ The system was finally constructed and solvated with tleap of AMBER package and was solvated on a truncated octahedral box of TIP3P^47,48^ water molecules, and the proper numbers of chlorine ions were added to achieve charge neutrality. Each system was then minimized, combining 5000 steps of steepest descent, followed by 25,000 steps of conjugate gradient, while the atoms of ligand and backbone atoms were restrained with a harmonic potential (30 kcal·mol^–1^ Å^–1^). Then, the systems were heated with Langevin thermostat in the NVT ensemble from 100 to 300 K in one stage of 120 ps using SHAKE^49^ with a time step of 2 fs. Temperature was controlled with a Langevin thermostat and a collision frequency of 5 ps^–1^. Then, an NPT relaxation was performed to equilibrate the system, reducing the harmonic potential from 20 to 15 kcal·mol^–1^ Å^–1^ and then to 0 in six stages of 2 ns (from 15 kcal·mol^–1^ Å^–1^ to 0). Finally, five 200 ns NVT independent MD simulations were performed for each system with the CUDA accelerated version of PMEMD.^50^ The stability of NVT simulations was monitored through the analysis of heavy atoms RMSD and RMSF (see Figures S16 to S25).

### Thermodynamic Integration Calculations

The final equilibrated structures for MD calculations of all complexes were used as starting points for thermodynamic integration (TI) calculations. The Amber TI protocol^51^ was employed to calculate the relative binding free energy changes between ligands in different CRBN–neosubstrate complexes. Specifically, we carried out the alchemical transformation of Thl to the four different ones presented in Figure 2 in the ternary complexes with both SALL4 and IKZF1, considering two possible poses, resulting in a total of 16 transformations.

The relative binding free energy differences (ΔΔ*G*_bind_) were determined from the mean values of five replicas for each transformation, both in aqueous solution and in the protein environment (see Figure S1 and Tables S1 and S2). Each transformation was defined by a coupling parameter, λ, changing from 0 to 1. For each system, the initial configuration for each of the five transformations was equilibrated at a λ value of 0.5. In each replica, 9 λ values were simulated (0.01592, 0.08198, 0.19331, 0.33787, 0.5, 0.66213, 0.80669, 0.91802, and 0.98408) and sampled during 40 ns per window. Replica exchange between adjacent λ values was attempted to improve conformational sampling. The convergence of the transformations was tracked by monitoring the behavior of ΔΔG_bind_ along the simulation time, as shown in Figures S14 and S15.

## Supplementary Material



## Data Availability

Initial structures for all molecular dynamic simulations and thermodynamic integration calculations, together with the corresponding input files and execution scripts used to perform these computations, are provided. In addition, a comprehensive set of Python scripts is included to compute and visualize structural descriptors such as root-mean-square deviation (RMSD), root-mean-square fluctuation (RMSF), and hydrogen-bonding profiles for all simulated systems, all available in 10.5281/zenodo.18312802.

## Notes

Supporting Figures S1 to S22 and Tables S1 and S2 with all the result of thermodynamic integrations (PDF)
