# Supplementary material for: Binding Modes of Thalidomide Derivatives in Cereblon–Neosubstrate Complexes Revealed by Molecular Dynamics and Free Energy Calculations
Source: ACS Omega. 2026 Jul 13;11(29):44515–25. doi: 10.1021/acsomega.6c06210 (PMC13425495; doi:10.1021/acsomega.6c06210)
Supplement: Supplementary file 1 [file ao6c06210_si_001.pdf]

Binding Modes of Thalidomide Derivatives in Cereblon–Neosubstrate Complexes Revealed by Molecular Dynamics and Free Energy Calculations

Verónica Martín,[1] Milorad Andjelkovic,[2] Carmen Barrientos,[1*] Iker Leon,[1,3*] Iñaki Tuñón[2*]

[1] Departamento de Química Física y Química Inorgánica, Universidad de Valladolid, 47011 Valladolid, Spain
[2] Departament de Quìmica Fisìca, Universitat de València, 46100 Burjassot, Spain
[3] Grupo de Espectroscopía Molecular (GEM), Edificio Quifima, Laboratorios de Espectroscopia y Bioespectroscopia, Unidad Asociada CSIC, Parque Científico UVa, Universidad de Valladolid, 47011 Valladolid, Spain

Figure S1	Hydrogen bond heatmap between CRBN, SALL4 and Pom (in and out) and a representative snapshot.	S3
Figure S2	Hydrogen bond heatmap between CRBN, IKZF1 and Pom (in and out) and a representative snapshot	S4
Figure S3	Scheme of alchemical transformations	S5
Table S1	Details of alchemical free energy calculations of the complex with SALL4	S6
Table S2	Details of alchemical free energy calculations of the complex with IKZF1	S8
Figure S4	Hydrogen bonds between chains: CRBN-SALL4 and CRBN-IKZF1	S10
Figure S5	Hydrogen bond heatmap between CRBN, SALL4 and 4ht (in or out) and a representative snapshot	S11
Figure S6	Hydrogen bond heatmap between CRBN, IKZF1 and 4ht (in and out) and a representative snapshot	S12
Figure S7	Hydrogen bond heatmap between CRBN, SALL4 and 5ht (in and out) and a representative snapshot	S13
Figure S8	Hydrogen bond heatmap between CRBN, IKZF1 and 5ht (in and out) and a representative snapshot	S14
Figure S9	Radial function of residue GLU 377 in complexes with SALL4. Comparison between Pom and Len	S15
Figure S10	Radial function of residue GLU 377 in complexes with IKZF1. Comparison between Pom and Len	S15
Figure S11	Hydrogen bond heatmap between CRBN, SALL4 and Len (in and out) and a representative snapshot	S16
Figure S12	Hydrogen bond heatmap between CRBN, IKZF1 and Len (in and out) and a representative snapshot	S17
Figure S13	Overlap between x-ray and simulated structure of the ternary complex CRBN-SALL4-5ht	S18
Figure S14	Convergence of the TI calculated value for the free energy versus simulation time in the ternary complex CRBN-Pom-SALL4	S18
Figure S15	Convergence of the TI calculated value for the free energy versus simulation time in the ternary complex CRBN-Pom-IKZF1	S19
Figure S16	Plot of the RMSD values of Pomalidomide in ternary complex	S19
Figure S17	Plot of the RMSD values of Thalidomide in ternary complex	S20

Figure S18	Plot of the RMSD values of 4-Thalidomide in ternary complex	S20
Figure S19	Plot of the RMSD values of 5-Thalidomide in ternary complex	S21
Figure S20	Plot of the RMSD values of Lenalidomide in ternary complex	S21
Figure S21	Plot of the RMSF values of Pomalidomide in ternary complex	S22
Figure S22	Plot of the RMSF values of Thalidomide in ternary complex	S22
Figure S23	Plot of the RMSF values of 4-Hidroxythalidomide in ternary complex	S23
Figure S24	Plot of the RMSF values of 5-Hidroxythalidomide in ternary complex	S23
Figure S25	Plot of the RMSF values of Lenalidomide in ternary complex	S24

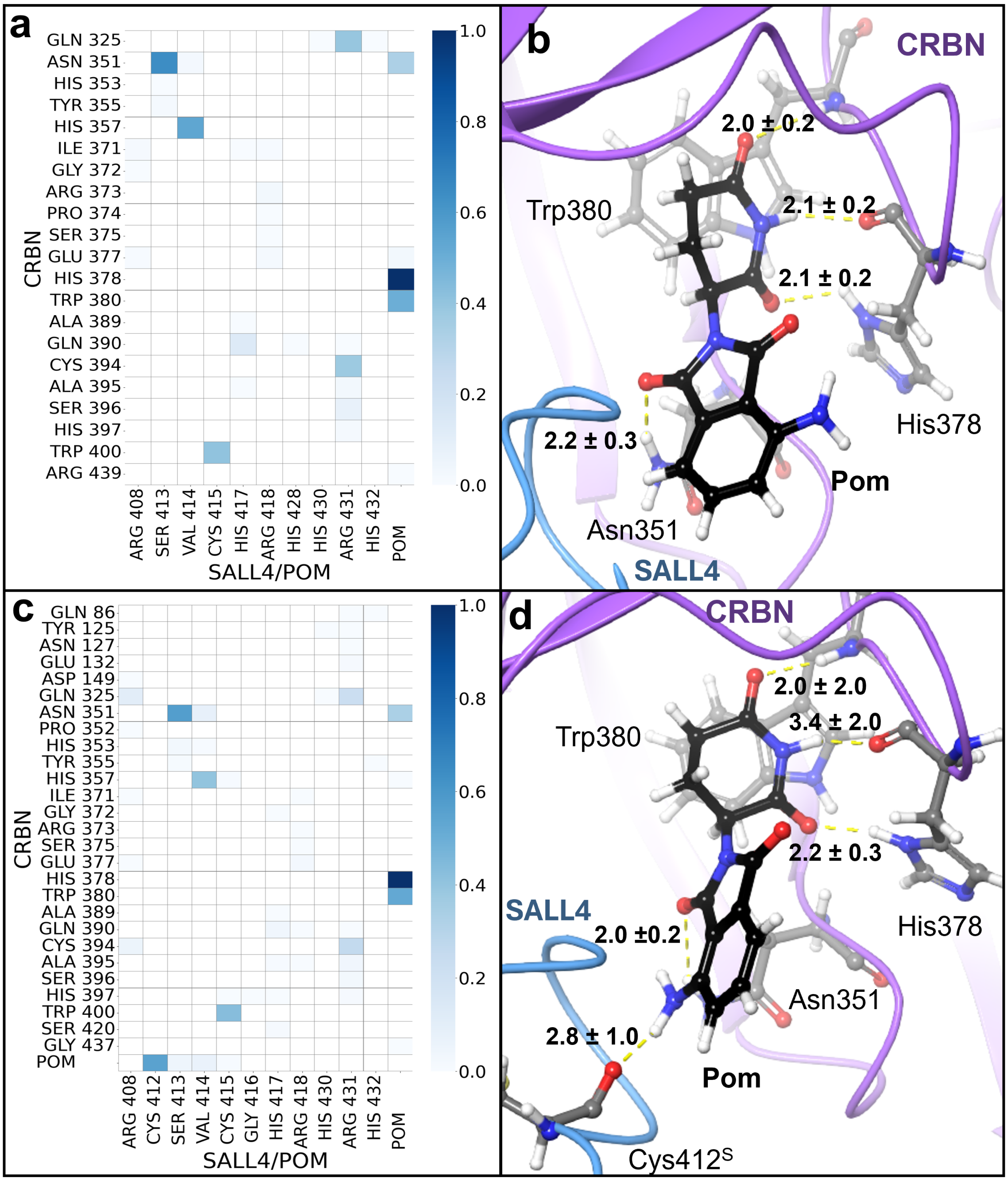

Figure S1 a) Hydrogen bond heatmap between CRBN, SALL4 and Pom in the "out" pose; **b)** Representative snapshot of Pom in complex with CRBN-SALL4, along with some average hydrogen bond distances in the "out" pose (in Angstroms); **c)** Hydrogen bond graph between CRBN, SALL4 and Pom in the "in" pose; **d)** Representative snapshot of Pom in complex with CRBN and SALL4, along with the average hydrogen bond distances (in Angstroms) in the "in" pose.

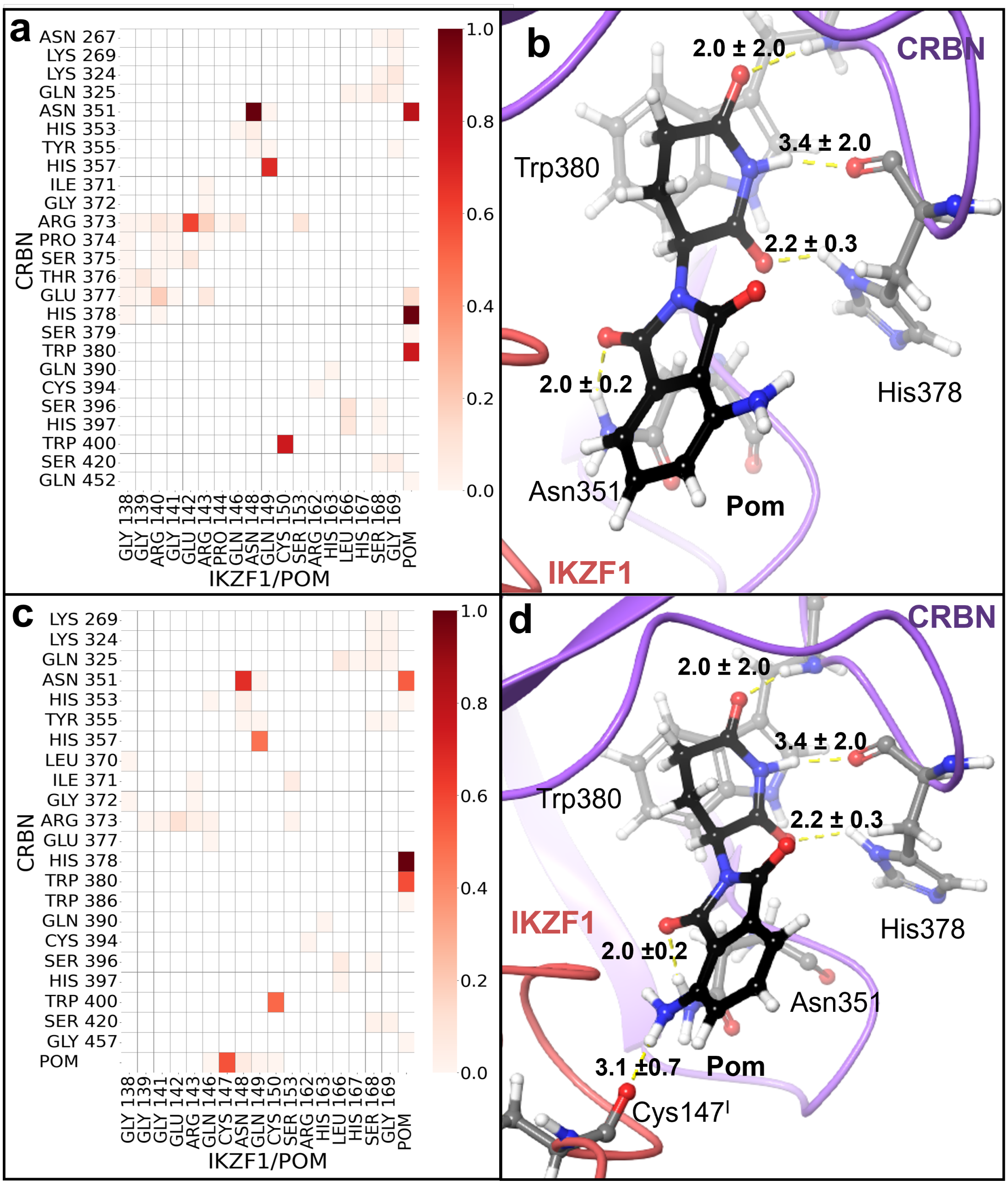

Figure S2 a) Hydrogen bond heatmap between CRBN, IKZF1 and Pom in the "out" pose; **b)** Representative snapshot of Pom in complex with CRBN-IKZF1, along with some average hydrogen bond distances (in Angstroms) in the "out" pose; **c)** Hydrogen bond graph between CRBN, IKZF1 and Pom in the "in" pose; **d)** Representative snapshot of Pom in complex with CRBN and IKZF1, along with the average hydrogen bond distances (in Angstroms) in the "in" pose.

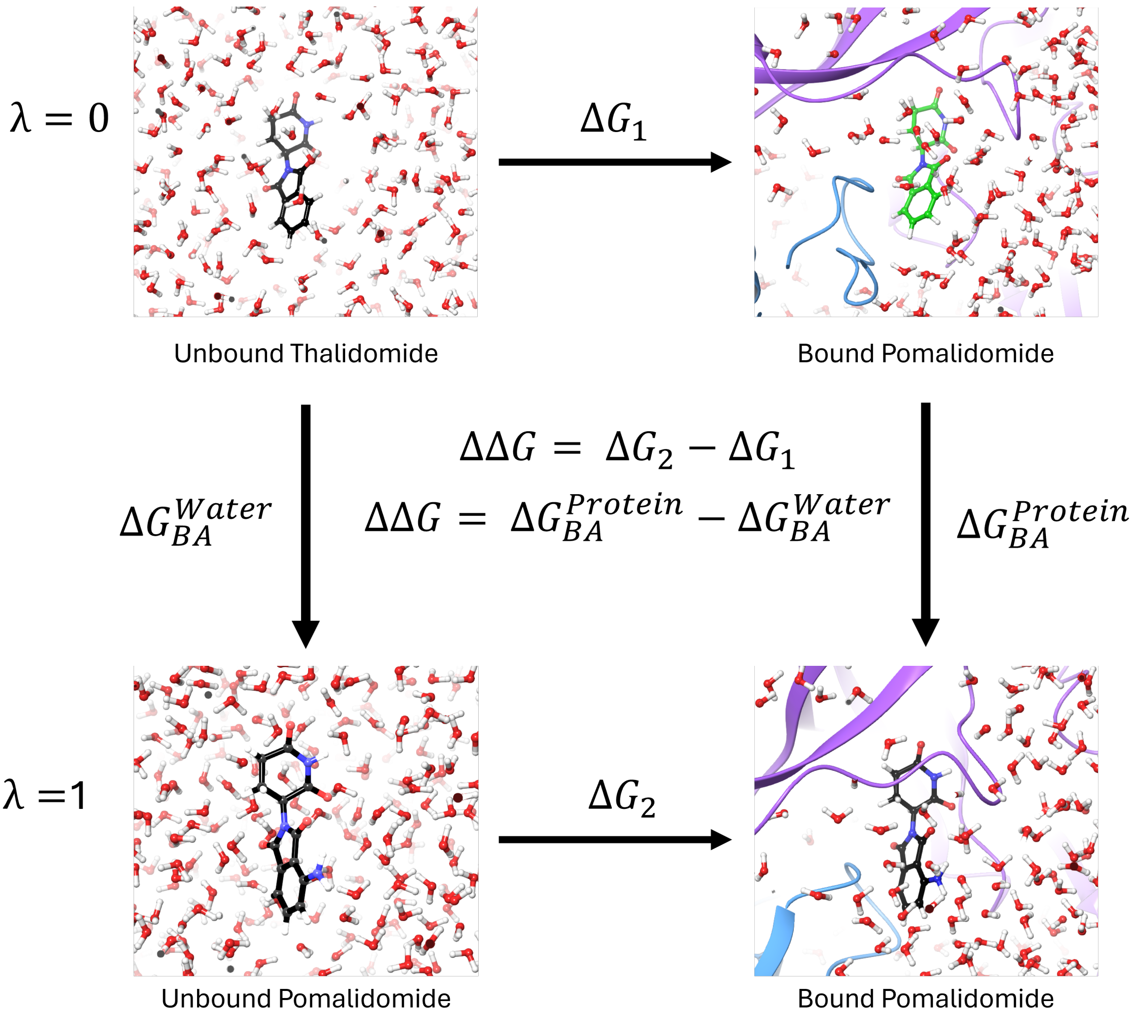

Figure S3. Thermodynamic cycle for alchemical transformation. The hydrogen of the thalidomide is replaced by the amino group of the pomalidomide. This method has been followed to obtain all the transformations in both X-ray structures tested.

Table S1. Free energy changes associated with alchemical transformations performed in aqueous and protein environments for the CRBN-ligand-SALL4 complex. Free energy values (in kcal·mol^{-1}) were estimated using TI and each average value is given with the corresponding standard deviation (std).

Thalidomide	to	Pom in	ΔΔG(kcal·mol^{-1})	1.93	± 0.12
System	Replica	ΔΔG(kcal·mol^{-1})	System	Replica	ΔΔG(kcal·mol^{-1})
Aqueous	1	-53.23	Protein	1	-51.17
Aqueous	2	-53.23	Protein	2	-51.50
Aqueous	3	-53.23	Protein	3	-51.17
Aqueous	4	-53.24	Protein	4	-51.30
Aqueous	5	-53.23	Protein	5	-51.33
Mean		-53.23	Mean		-51.29
Std		0.01	std		0.12
Thalidomide	to	Pom out	ΔΔG(kcal·mol^{-1})	-0.13	± 0.03
System	Replica	ΔΔG(kcal·mol^{-1})	System	Replica	ΔΔG(kcal·mol^{-1})
Aqueous	1	-53.26	Protein	1	-53.43
Aqueous	2	-53.25	Protein	2	-53.35
Aqueous	3	-53.26	Protein	3	-53.40
Aqueous	4	-53.27	Protein	4	-53.38
Aqueous	5	-53.26	Protein	5	-53.41
Mean		-53.26	Mean		-53.39
Std		0.01	std		0.03
Thalidomide	to	4ht in	ΔΔG(kcal·mol^{-1})	1.2	± 0.08
System	Replica	ΔΔG(kcal·mol^{-1})	System	Replica	ΔΔG(kcal·mol^{-1})
Aqueous	1	-29.08	Protein	1	-27.87
Aqueous	2	-29.10	Protein	2	-27.79
Aqueous	3	-29.09	Protein	3	-28.04
Aqueous	4	-29.10	Protein	4	-27.91
Aqueous	5	-29.10	Protein	5	-27.86
Mean		-29.09	Mean		-27.89
Std		0.01	std		0.08
Thalidomide	to	4ht out	ΔΔG(kcal·mol^{-1})	-0.01	± 0.02
System	Replica	ΔΔG(kcal·mol^{-1})	System	Replica	ΔΔG(kcal·mol^{-1})
Aqueous	1	-29.10	Protein	1	-29.08
Aqueous	2	-29.12	Protein	2	-29.12
Aqueous	3	-29.09	Protein	3	-29.14
Aqueous	4	-29.10	Protein	4	-29.12
Aqueous	5	-29.09	Protein	5	-29.09
Mean		-29.10	Mean		-29.11
Std		0.01	std		0.02
Thalidomide	to	5ht in	ΔΔG(kcal·mol^{-1})	1.33	± 0.07
System	Replica	ΔΔG(kcal·mol^{-1})	System	Replica	ΔΔG(kcal·mol^{-1})
Aqueous	1	-21.37	Protein	1	-20.02
Aqueous	2	-21.36	Protein	2	-20.08
Aqueous	3	-21.36	Protein	3	-19.96
Aqueous	4	-21.36	Protein	4	-19.96
Aqueous	5	-21.37	Protein	5	-20.16

Mean		-21.36	Mean		-20.04
Std		0.01	std		0.08
Thalidomide	to	5ht out	ΔΔG(kcal·mol^{-1})	0.05	± 0.04
System	Replica	ΔΔG(kcal·mol^{-1})	System	Replica	ΔΔG(kcal·mol^{-1})
Aqueous	1	-21.35	Protein	1	-21.29
Aqueous	2	-21.36	Protein	2	-21.29
Aqueous	3	-21.36	Protein	3	-21.31
Aqueous	4	-21.35	Protein	4	-21.37
Aqueous	5	-21.36	Protein	5	-21.25
Mean		-21.36	Mean		-21.30
Std		0.01	std		0.04
Thalidomide	to	Len in	ΔΔG(kcal·mol^{-1})	5.13	± 0.42
System	Replica	ΔΔG(kcal·mol^{-1})	System	Replica	ΔΔG(kcal·mol^{-1})
Aqueous	1	11.42	Protein	1	16.26
Aqueous	2	11.42	Protein	2	16.16
Aqueous	3	11.41	Protein	3	16.68
Aqueous	4	11.40	Protein	4	16.35
Aqueous	5	11.45	Protein	5	17.31
Mean		11.42	Mean		16.55
Std		0.02	std		0.42
Thalidomide	to	Len out	ΔΔG(kcal·mol^{-1})	-2.32	± 0.12
System	Replica	ΔΔG(kcal·mol^{-1})	System	Replica	ΔΔG(kcal·mol^{-1})
Aqueous	1	11.92	Protein	1	9.70
Aqueous	2	11.81	Protein	2	9.56
Aqueous	3	11.87	Protein	3	9.66
Aqueous	4	12.12	Protein	4	9.59
Aqueous	5	11.96	Protein	5	9.58
Mean		11.94	Mean		9.62
Std		0.10	std		0.05

Table S2. Free energy changes associated with alchemical transformations performed in aqueous and protein environments for the CRBN-ligand-IKZF1 complex. Free energy values (in kcal·mol^{-1}) were estimated using TI and each average value is given with the corresponding standard deviation (std).

Thalidomide	to	Pom in	ΔΔG(kcal·mol^{-1})	2.95	± 0.04
System	Replica	ΔΔG(kcal·mol^{-1})	System	Replica	ΔΔG(kcal·mol^{-1})
Aqueous	1	-53.23	Protein	1	-50.23
Aqueous	2	-53.24	Protein	2	-50.26
Aqueous	3	-53.22	Protein	3	-50.24
Aqueous	4	-53.22	Protein	4	-50.32
Aqueous	5	-53.22	Protein	5	-50.32
Mean		-53.23	Mean		-50.27
Std		0.01	std		0.04
Thalidomide	to	Pom out	ΔΔG(kcal·mol^{-1})	-0.47	± 0.02
System	Replica	ΔΔG(kcal·mol^{-1})	System	Replica	ΔΔG(kcal·mol^{-1})
Aqueous	1	-53.26	Protein	1	-53.70
Aqueous	2	-53.25	Protein	2	-53.72
Aqueous	3	-53.26	Protein	3	-53.71
Aqueous	4	-53.27	Protein	4	-53.73
Aqueous	5	-53.26	Protein	5	-53.76
Mean		-53.26	Mean		-53.73
Std		0.01	std		0.02
Thalidomide	to	4ht in	ΔΔG(kcal·mol^{-1})	2.29	± 0.08
System	Replica	ΔΔG(kcal·mol^{-1})	System	Replica	ΔΔG(kcal·mol^{-1})
Aqueous	1	-35.52	Protein	1	-33.37
Aqueous	2	-35.52	Protein	2	-33.21
Aqueous	3	-35.54	Protein	3	-33.23
Aqueous	4	-35.50	Protein	4	-33.15
Aqueous	5	-35.51	Protein	5	-33.10
Mean		-35.52	Mean		-33.21
Std		0.01	std		0.08
Thalidomide	to	4ht out	ΔΔG(kcal·mol^{-1})	0.37	± 0.05
System	Replica	ΔΔG(kcal·mol^{-1})	System	Replica	ΔΔG(kcal·mol^{-1})
Aqueous	1	-35.53	Protein	1	-35.23
Aqueous	2	-35.55	Protein	2	-35.15
Aqueous	3	-35.53	Protein	3	-35.19
Aqueous	4	-35.50	Protein	4	-35.18
Aqueous	5	-35.55	Protein	5	-35.08
Mean		-35.53	Mean		-35.17
Std		0.02	std		0.05
Thalidomide	to	5ht in	ΔΔG(kcal·mol^{-1})	-0.21	± 0.02
System	Replica	ΔΔG(kcal·mol^{-1})	System	Replica	ΔΔG(kcal·mol^{-1})
Aqueous	1	-21.51	Protein	1	-21.73
Aqueous	2	-21.51	Protein	2	-21.69
Aqueous	3	-21.51	Protein	3	-21.74
Aqueous	4	-21.52	Protein	4	-21.73
Aqueous	5	-21.51	Protein	5	-21.74

Mean		-21.51	Mean		-21.72
Std		0.01	std		0.02
Thalidomide	to	5ht out	ΔΔG(kcal·mol^{-1})	-0.44	± 0.01
System	Replica	ΔΔG(kcal·mol^{-1})	System	Replica	ΔΔG(kcal·mol^{-1})
Aqueous	1	-21.50	Protein	1	-21.93
Aqueous	2	-21.49	Protein	2	-21.95
Aqueous	3	-21.49	Protein	3	-21.93
Aqueous	4	-21.50	Protein	4	-21.96
Aqueous	5	-21.49	Protein	5	-21.94
Mean		-21.49	Mean		-21.94
Std		0.01	std		0.01
Thalidomide	to	Len in	ΔΔG(kcal·mol^{-1})	4.12	± 0.31
System	Replica	ΔΔG(kcal·mol^{-1})	System	Replica	ΔΔG(kcal·mol^{-1})
Aqueous	1	11.85	Protein	1	15.78
Aqueous	2	11.82	Protein	2	16.34
Aqueous	3	11.84	Protein	3	15.84
Aqueous	4	11.40	Protein	4	15.85
Aqueous	5	12.00	Protein	5	15.69
Mean		11.78	Mean		15.90
Std		0.20	std		0.23
Thalidomide	to	Len out	ΔΔG(kcal·mol^{-1})	-3.96	± 0.15
System	Replica	ΔΔG(kcal·mol^{-1})	System	Replica	ΔΔG(kcal·mol^{-1})
Aqueous	1	11.84	Protein	1	7.98
Aqueous	2	11.82	Protein	2	8.03
Aqueous	3	11.85	Protein	3	7.66
Aqueous	4	11.89	Protein	4	7.96
Aqueous	5	12.00	Protein	5	7.99
Mean		11.88	Mean		7.92
Std		0.06	std		0.13

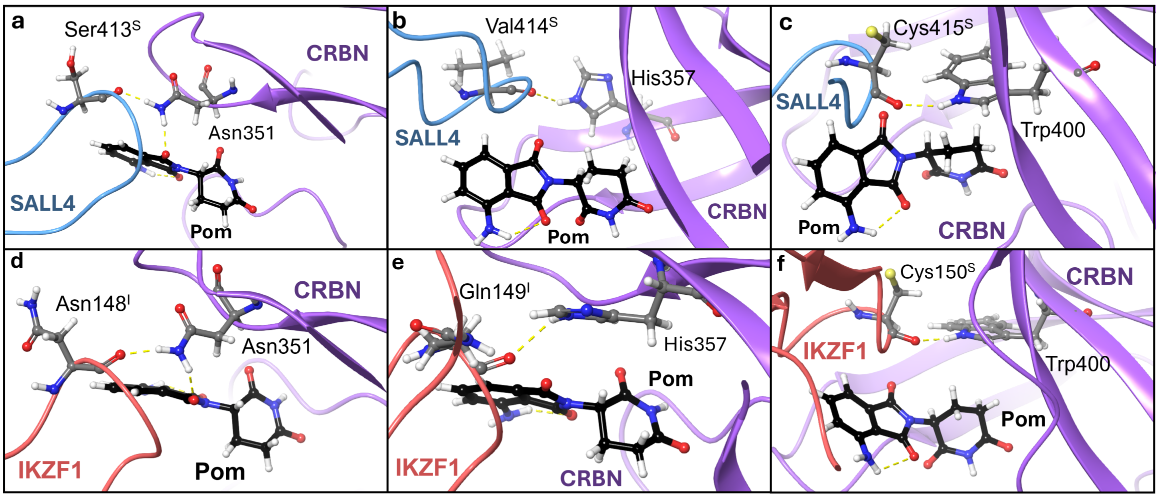

Figure S4. H-bonds between chains. Figures a), b) and c), represent the hydrogen bonds between protein CRBN and the neosubstrate SALL4 with Pom inserted in the binding site. Figures d) to f) represent the hydrogen bonds between the protein chains of CRBN and IKZF1 also with Pom in the binding site.

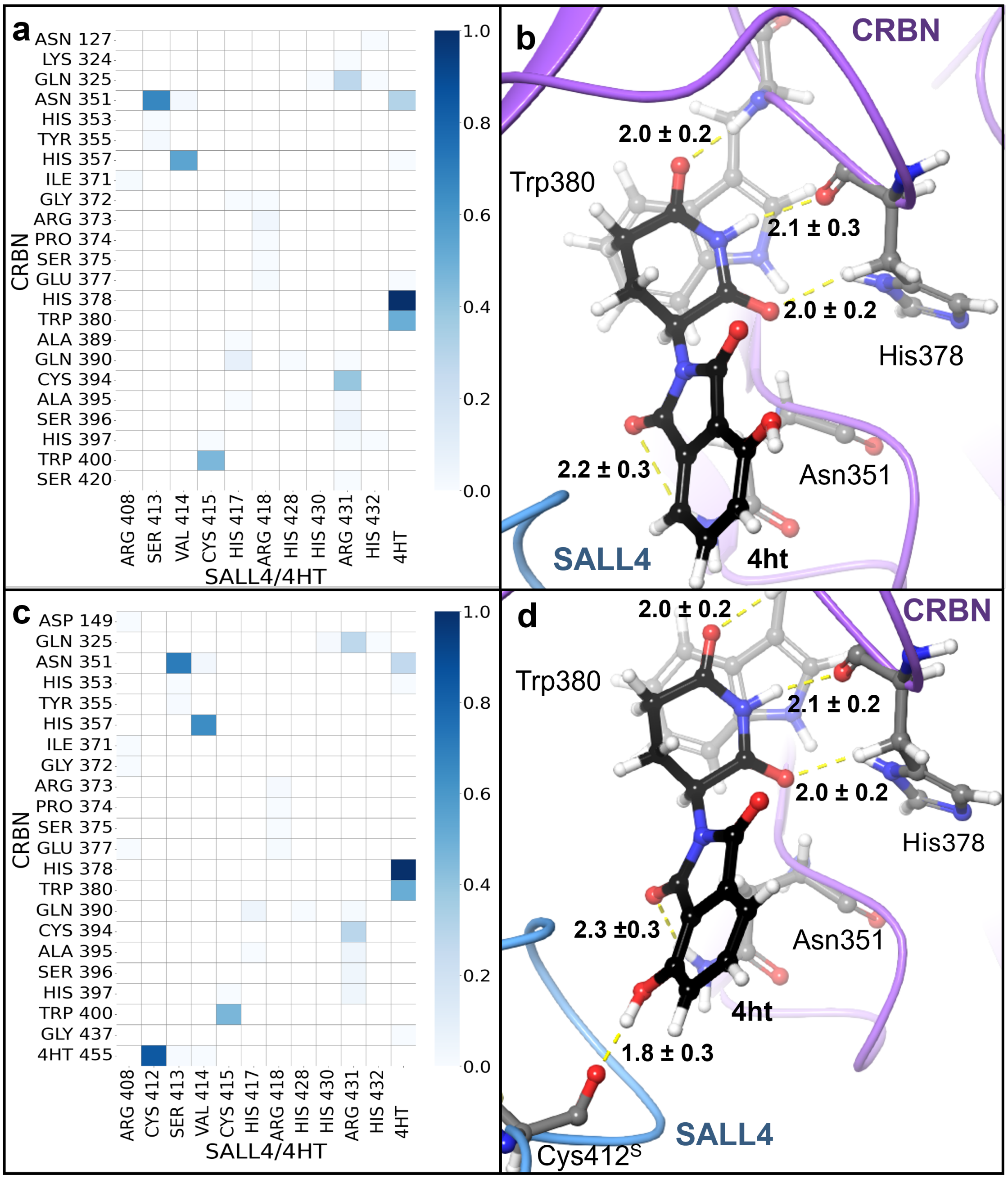

Figure S5 a) Hydrogen bond heatmap between CRBN, SALL4 and 4ht in the "out" pose; **b)** Representative snapshot of 4ht in complex with CRBN-SALL4, along with some average hydrogen bond distances (in Angstroms) in the "out" pose; **c)** Hydrogen bond graph between CRBN, SALL4 and 4ht in the "in" pose; **d)** Representative snapshot of 4ht in complex with CRBN and SALL4, along with the average hydrogen bond distances (in Angstroms) in the "in" pose .

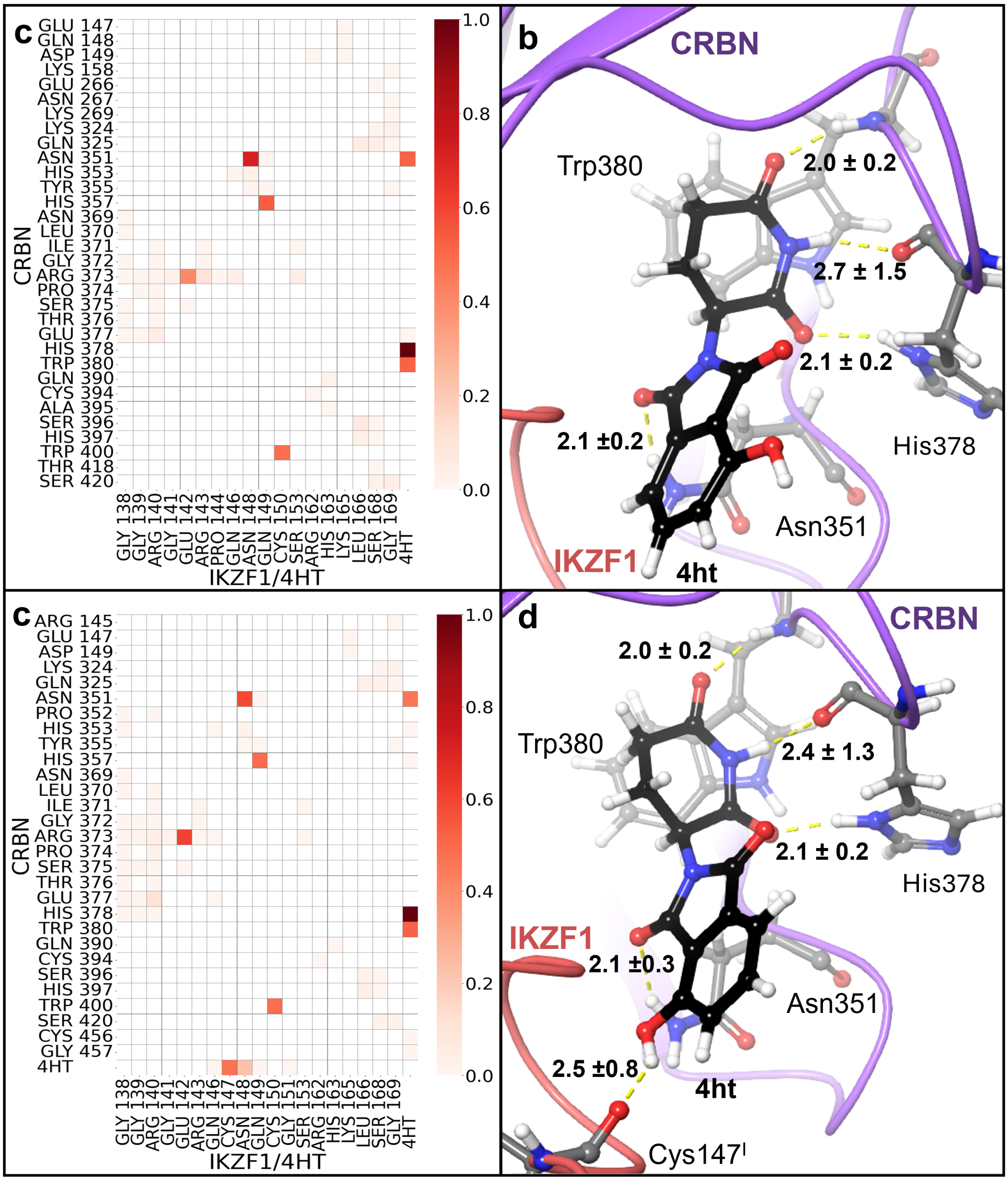

Figure S6 a) Hydrogen bond heatmap between CRBN, IKZF1 and 4ht in the “out” pose; **b)** Representative snapshot of 4ht in complex with CRBN-IKZF1, along with some average hydrogen bond distances (in Angstroms) in the “out” pose; **c)** Hydrogen bond graph between CRBN, IKZF1 and 4ht in the “in” pose; **d)** Representative snapshot of 4ht in complex with CRBN and IKZF1, along with the average hydrogen bond distances (in Angstroms) in the “in” pose.

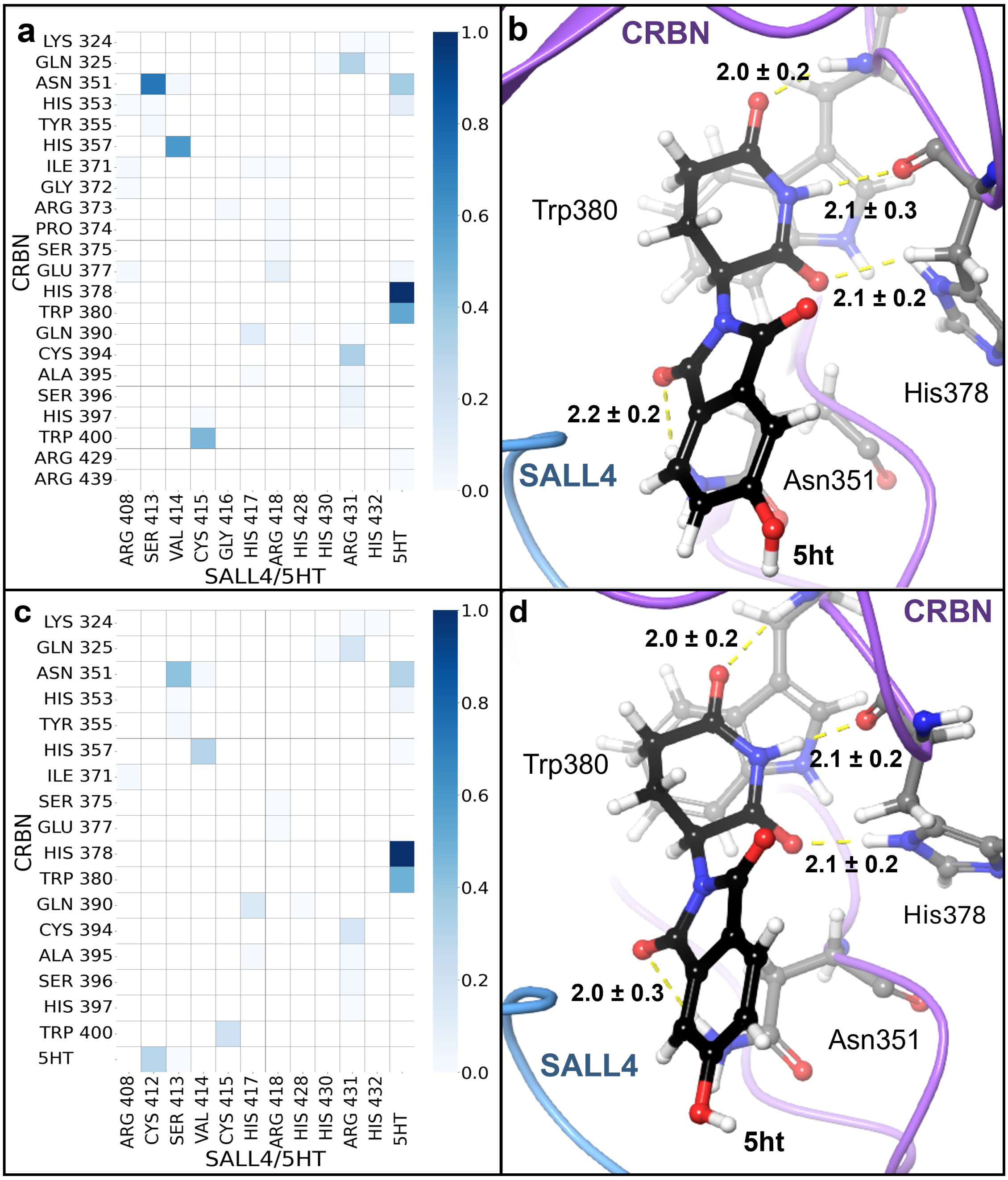

Figure S7 a) Hydrogen bond heatmap between CRBN, SALL4 and 5ht in the "out" pose; **b)** Representative snapshot of 5ht in complex with CRBN-SALL4, along with some average hydrogen bond distances (in Angstroms) in the "out" pose; **c)** Hydrogen bond graph between CRBN, SALL4 and 5ht in the "in" pose; **d)** Representative snapshot of 5ht in complex with CRBN and SALL4, along with the average hydrogen bond distances (in Angstroms) in the "in" pose.

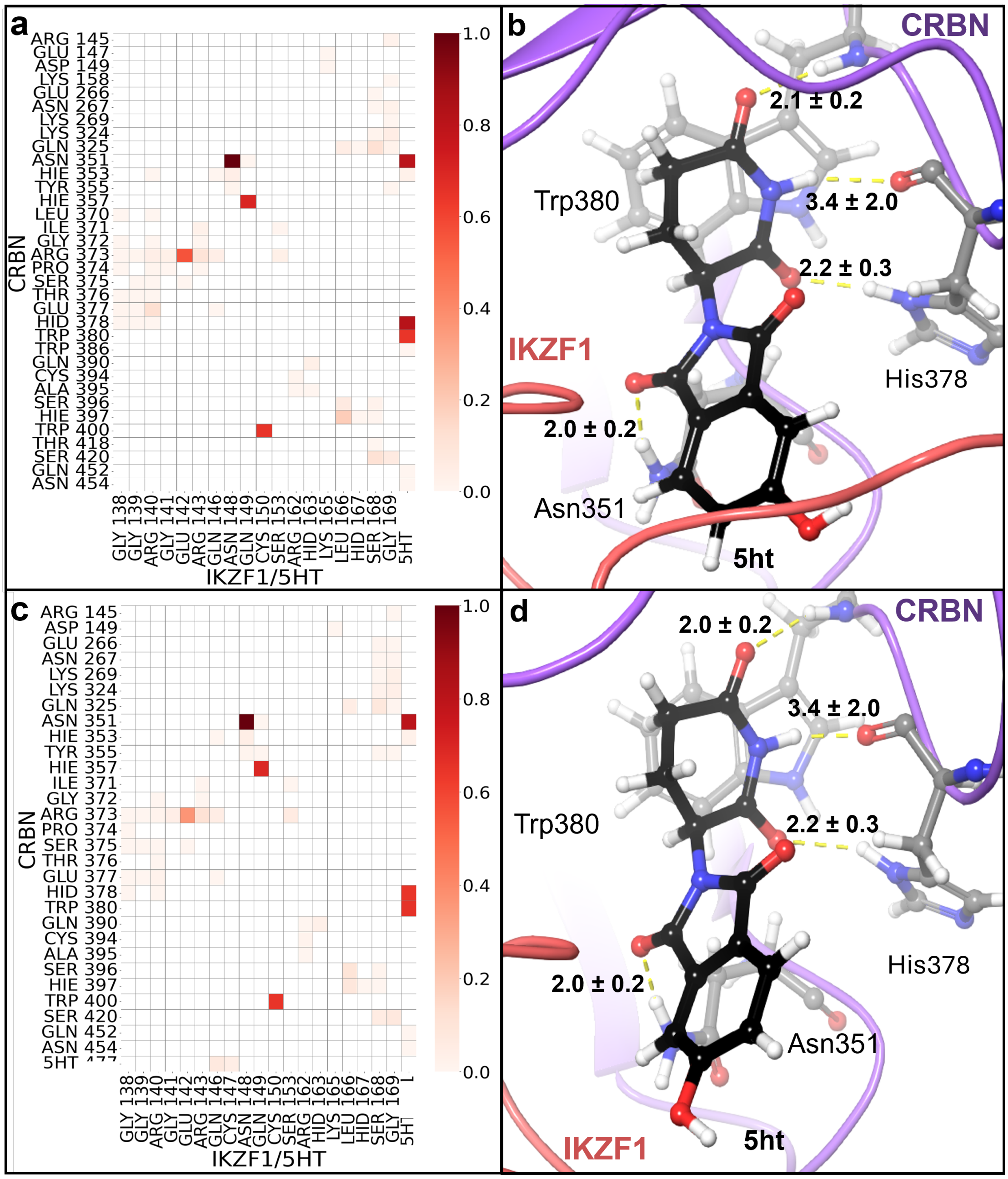

Figure S8 a) Hydrogen bond heatmap between CRBN, IKZF1 and 5ht in the "out" pose; **b)** Representative snapshot of 5ht in complex with CRBN-SALL4, along with some average hydrogen bond distances in the "out" pose; **c)** Hydrogen bond graph between CRBN, SALL4 and 5ht in the "in" pose; **d)** Representative snapshot of 5ht in complex with CRBN and IKZF1, along with the average hydrogen bond distances (in Angstroms) withIKZF1 in the "in" pose.

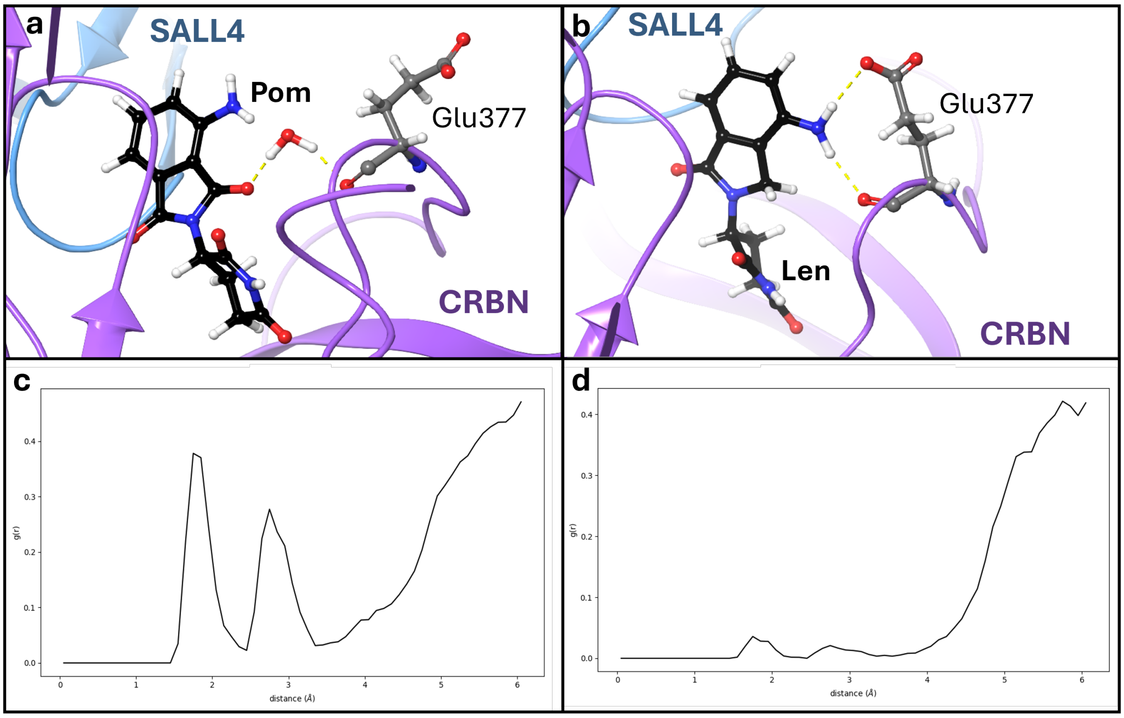

Figure S9 Radial distribution function of water oxygen atoms around the carbonyl oxygen atom of Glu377 in the complex with SALL4: **a)** Water mediated interaction between Pom and Glu377; **b**) Direct hydrogen bond interaction between Len and Glu377, **c**) Radial distribution function of water oxygen atoms around the carbonyl oxygen atom of Glu377 with pomalidomide as ligand, **d**) Radial distribution function of water oxygen atoms around the carbonyl oxygen atom of Glu377 with lenalidomide as ligand.

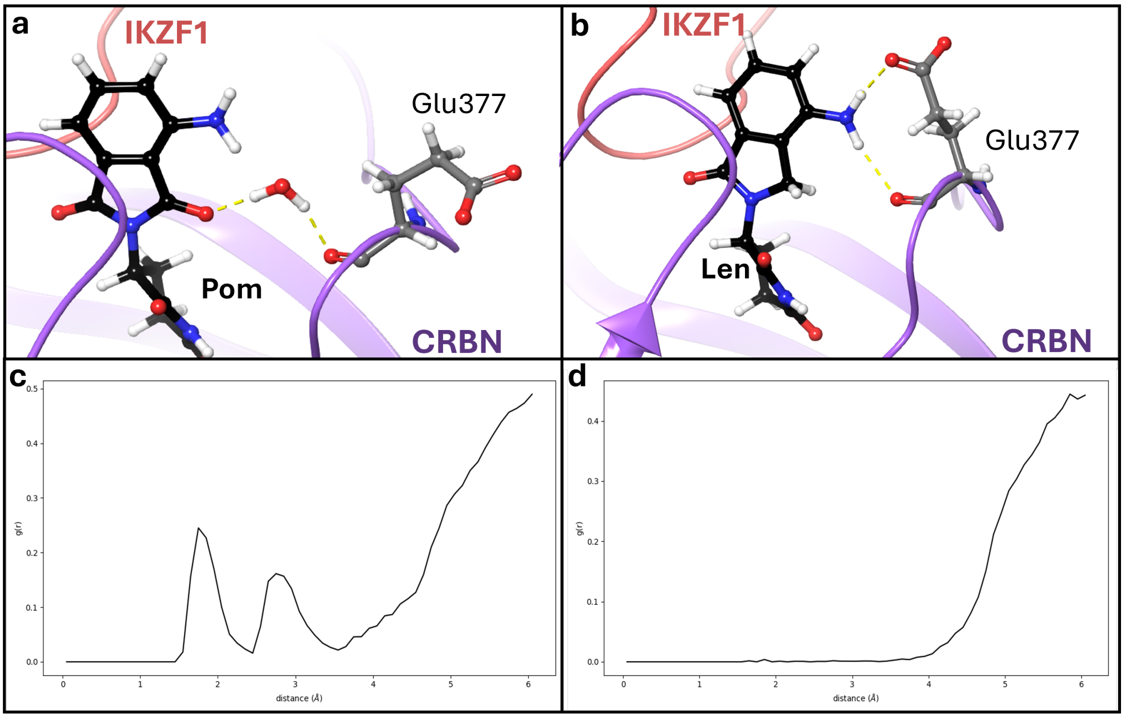

Figure S10 Radial distribution function of water oxygen atoms around the carbonyl oxygen atom of Glu377 in the complex with IKZF1: **a)** Water mediated interaction between Pom and Glu377; **b**) Direct hydrogen bond interaction between Len and Glu377, **c**) Radial distribution function of water oxygen atoms around the carbonyl oxygen atom of Glu377 with pomalidomide as ligand, **d**) Radial distribution function of water oxygen atoms around the carbonyl oxygen atom of Glu377 with lenalidomide as ligand.

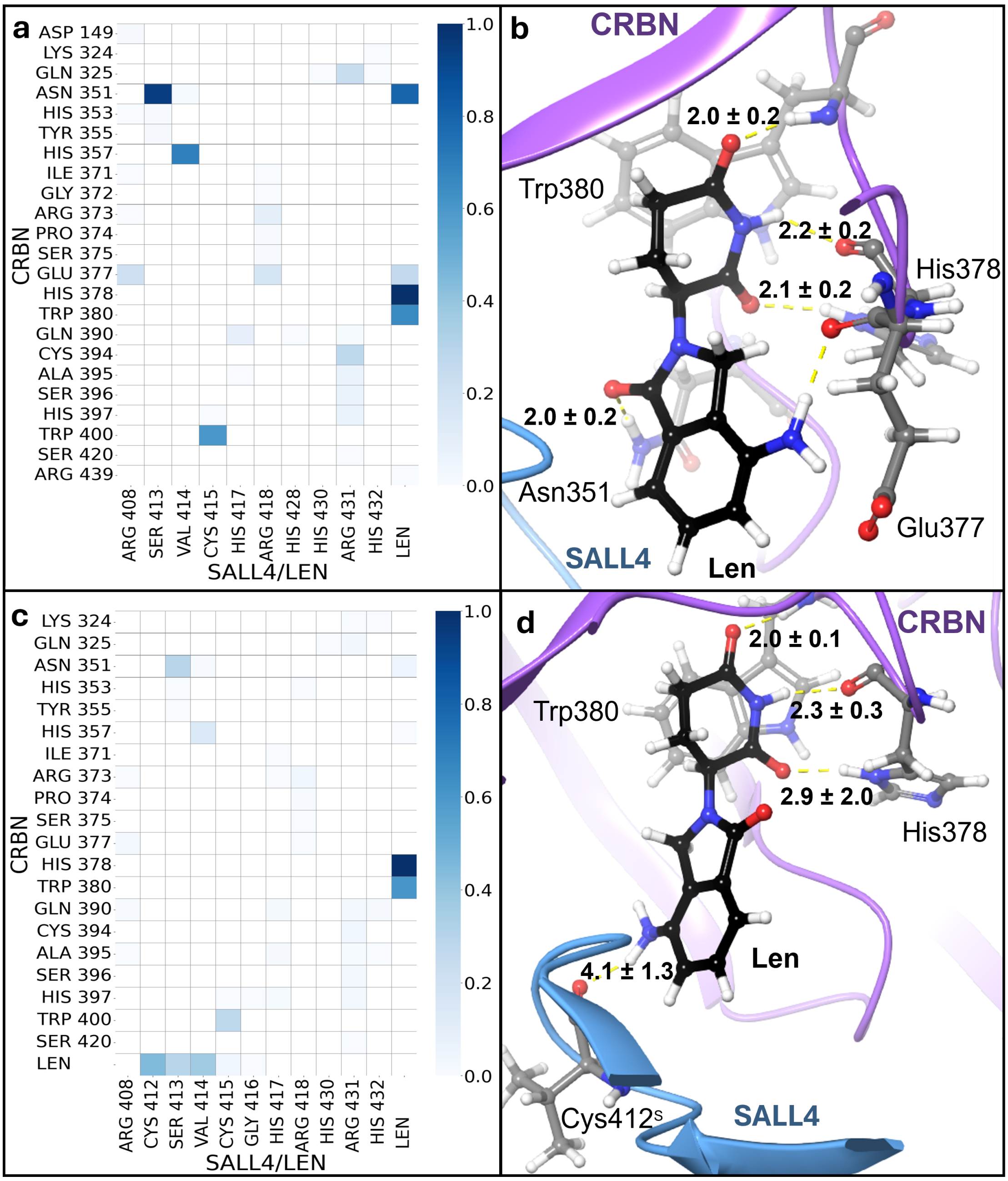

Figure S11 a) Hydrogen bond heatmap between CRBN, SALL4 and Len in the “out” pose; **b)** Representative snapshot of Len in complex with CRBN-SALL4, along with some average hydrogen bond distances (in Angstroms) in the “out” pose; **c)** Hydrogen bond graph between CRBN, SALL4 and Len in the “in” pose; **d)** Hydrogen bonds between Len and CRBN, along with the average hydrogen bond distances (in Angstroms) in the “in” pose.

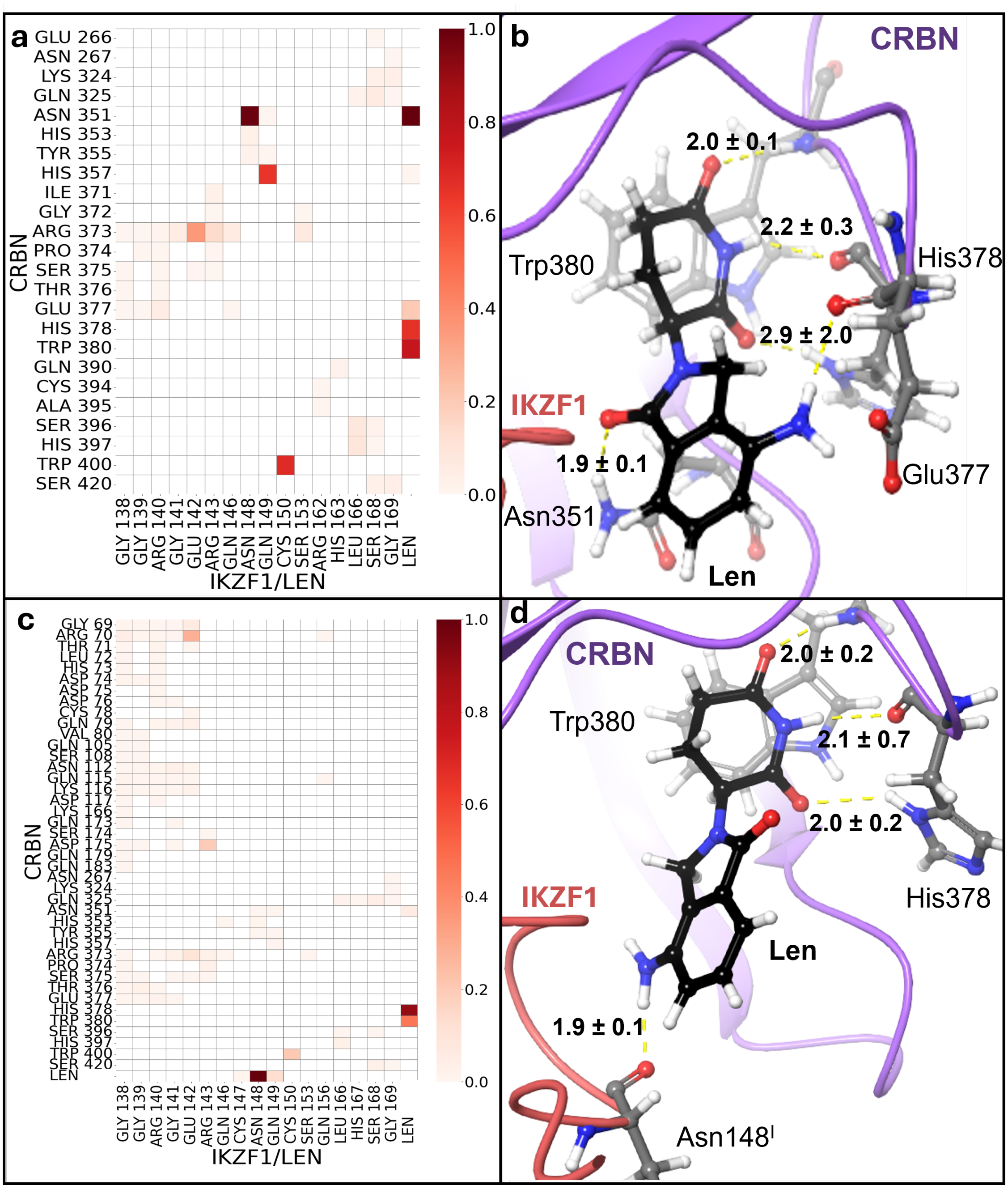

Figure S12 a) Hydrogen bond heatmap between CRBN, IKZF1 and Len in the "out" pose; **b)** Representative snapshot of Len in complex with CRBN-IKZF1, along with some average hydrogen bond distances (in Angstroms) in the "out" pose; **c)** Hydrogen bond graph between CRBN, IKZF1 and Len in the "in" pose; **d)** Hydrogen bonds between Len and CRBN, along with the average bond distances (in Angstroms) in the "in" pose.

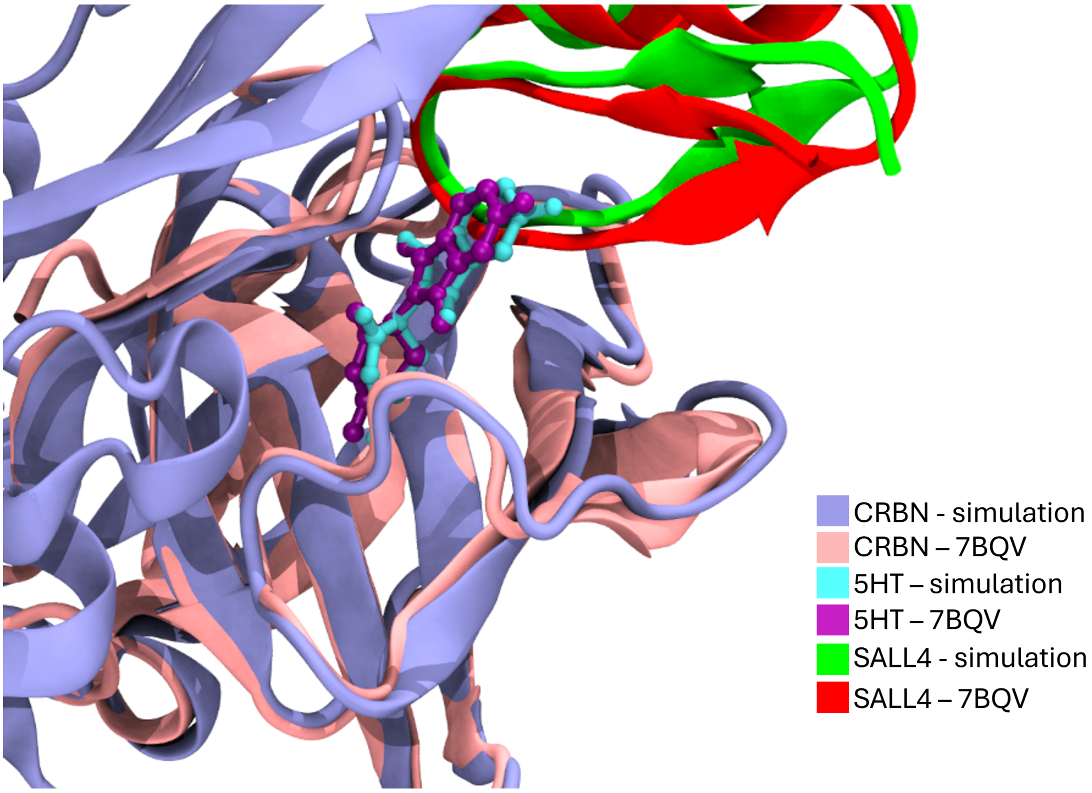

Figure S13. Overlay of the PDB structure 7BQV (CRBN in pink, ligand in purple and SALL4 in red) with a snapshot of the simulations of 5HT (CRBN in iceblue, ligand in blue and SALL4 in green) showing CRBN in the close conformation and the neosubstrate SALL4.

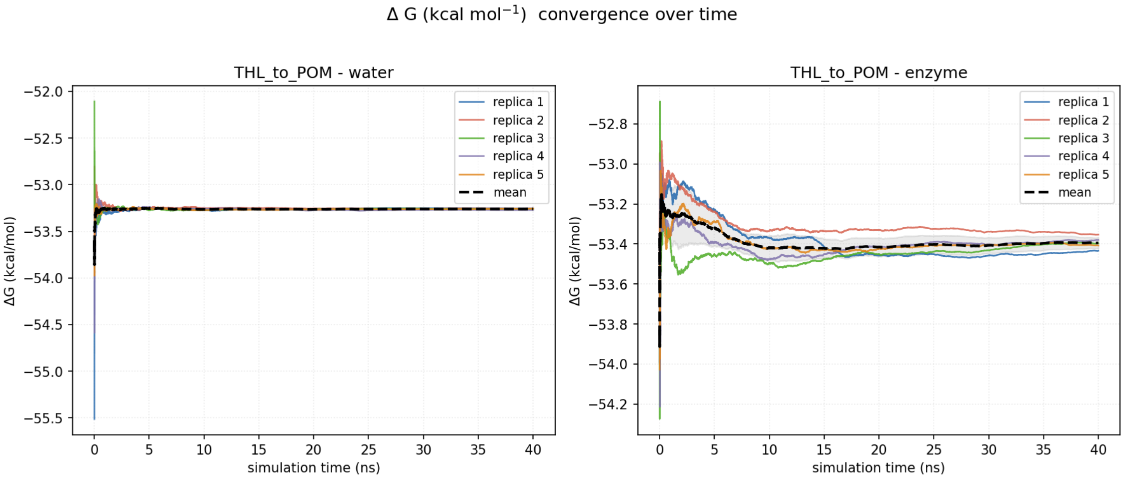

Figure S14. Variation of the value of ΔG for the transformation from thalidomide to pomalidomide in water (left) and in the complex with CRBN and SALL4 (right).

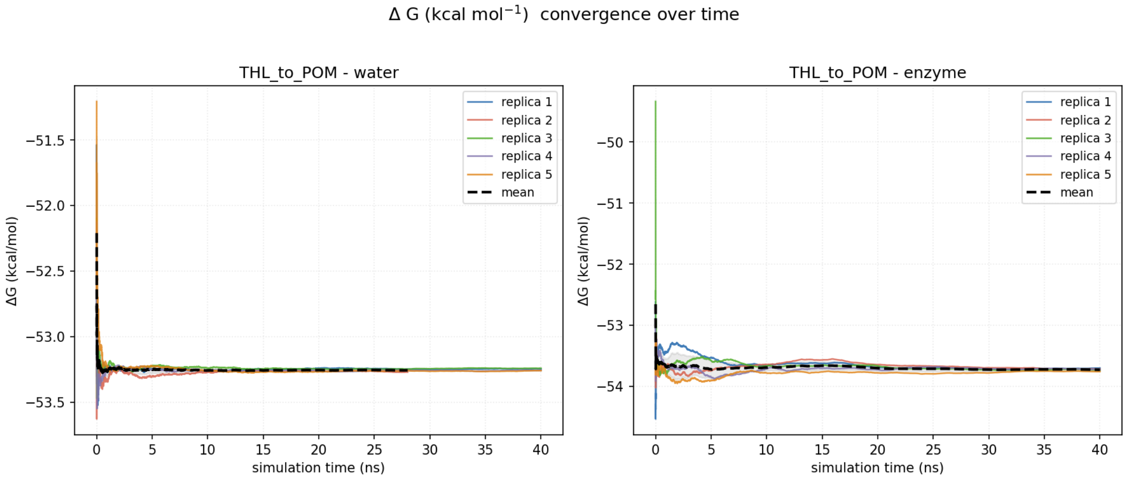

Figure S15. Variation of the value of ΔG for the transformation from thalidomide to pomalidomide in water (left) and in the complex with CRBN and IKZF1 (right).

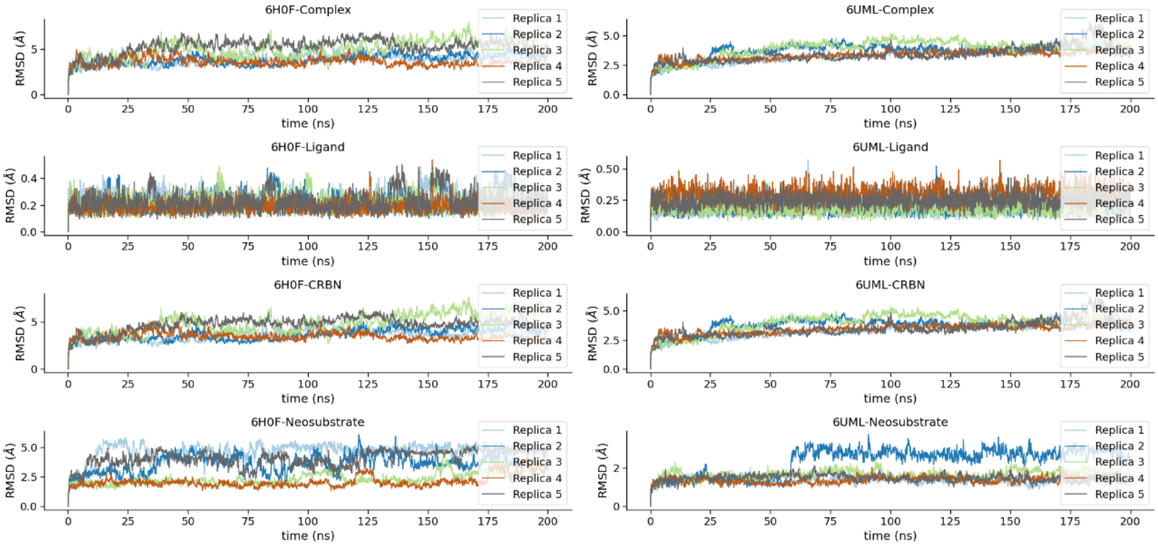

Figure S16 RMSD obtained during the five replicas of the simulations with pomalidomide in the pocket for complex, neosubstrate, ligand and CRBN protein. Left panels simulations with SALL4, right panels with IKZF1.

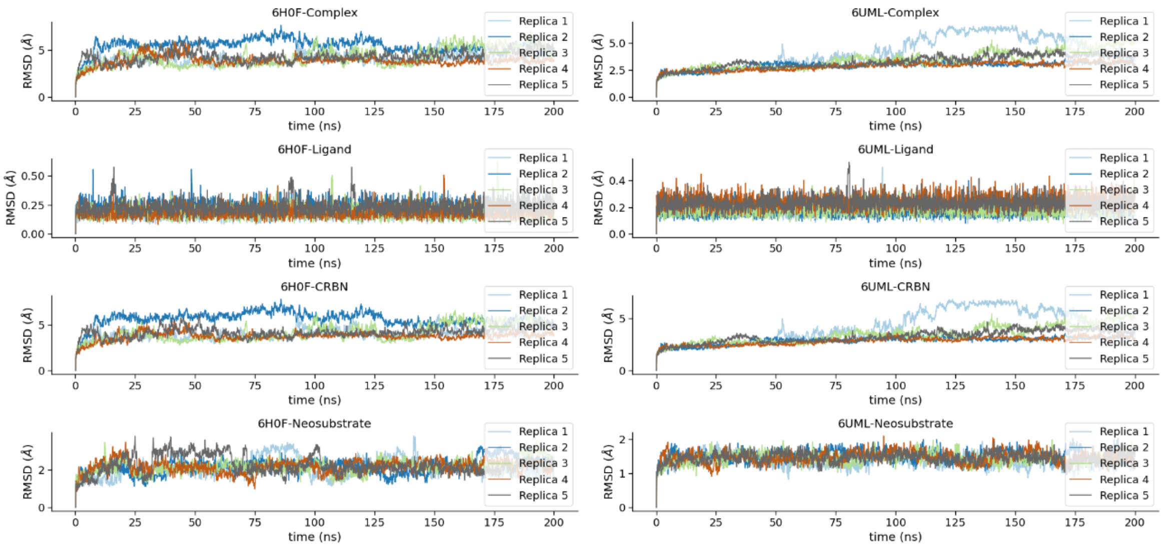

Figure S17 RMSD obtained during the five replicas of the simulations with thalidomide in the pocket for: Complex, neosubstrate, ligand and CRBN protein. Left panels simulations with SALL4, right panels with IKZF1.

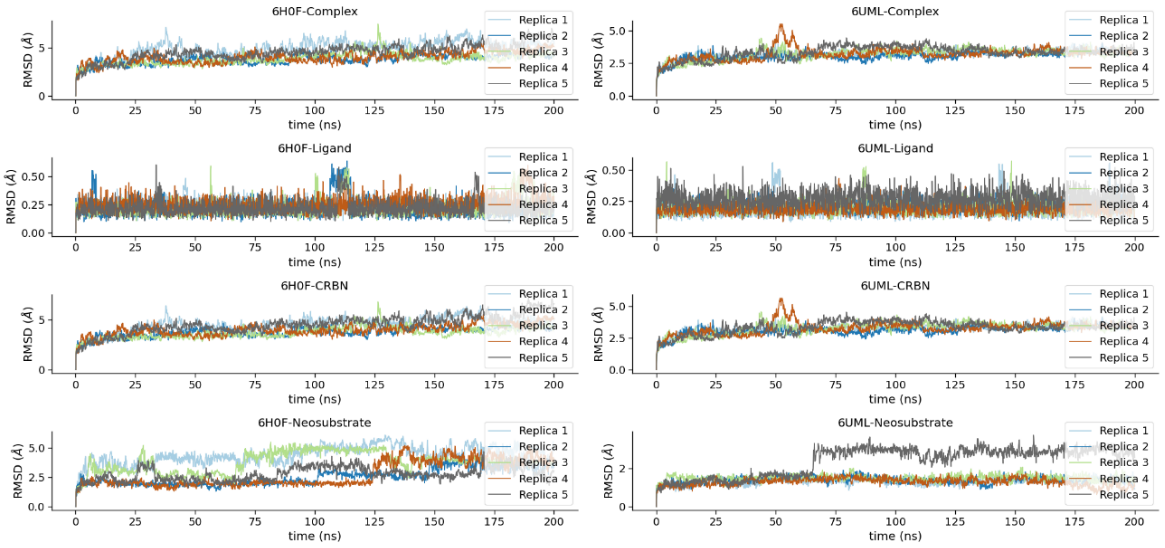

Figure S18 RMSD obtained during the five replicas of the simulations with 5-hydroxythalidomide in the pocket for complex, neosubstrate, ligand and CRBN protein. Left panels simulations with SALL4, right panels with IKZF1.

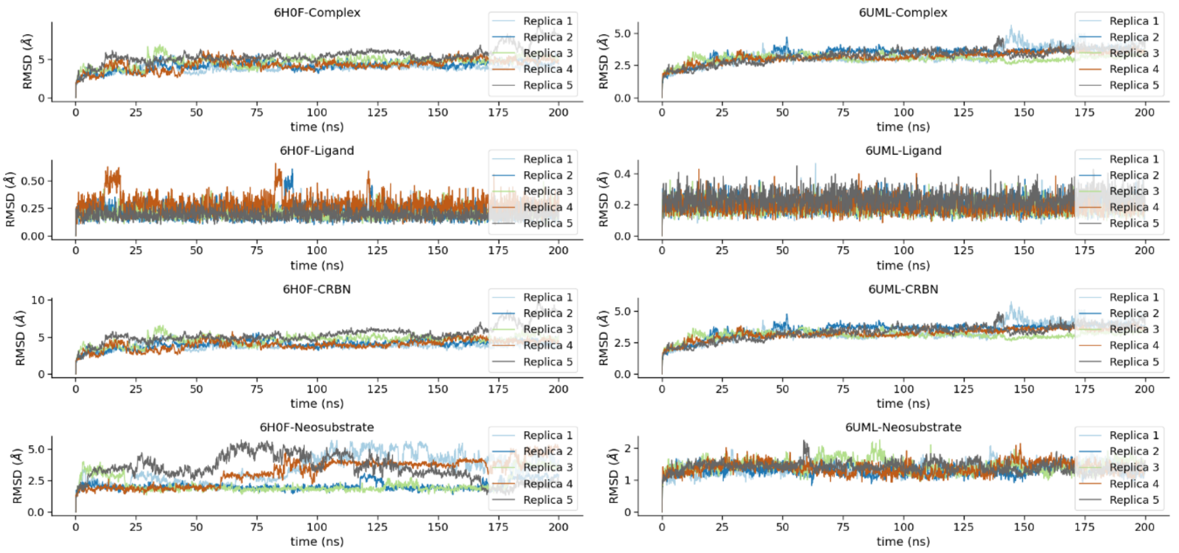

Figure S19 RMSD obtained during the five replicas of the simulations with 4-hydroxythalidomide in the pocket for complex, neosubstrate, ligand and CRBN protein. Left panels simulations with SALL4, right panels with IKZF1.

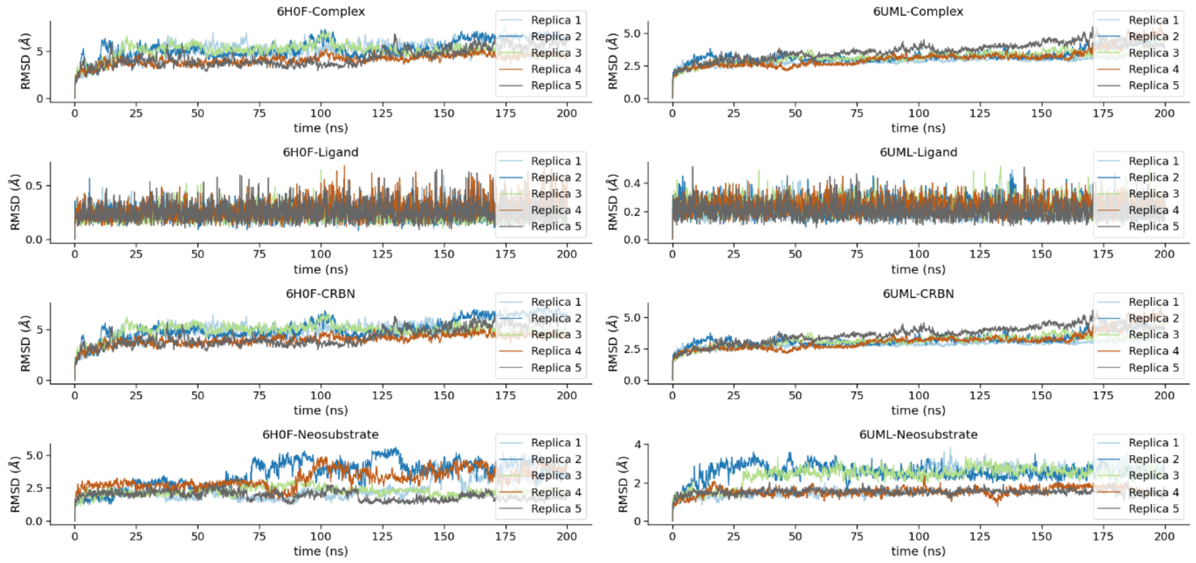

Figure S20 RMSD obtained during the five replicas of the simulations with lenalidomide in the pocket for complex, neosubstrate, ligand and CRBN protein. Left panels simulations with SALL4, right panels with IKZF1.

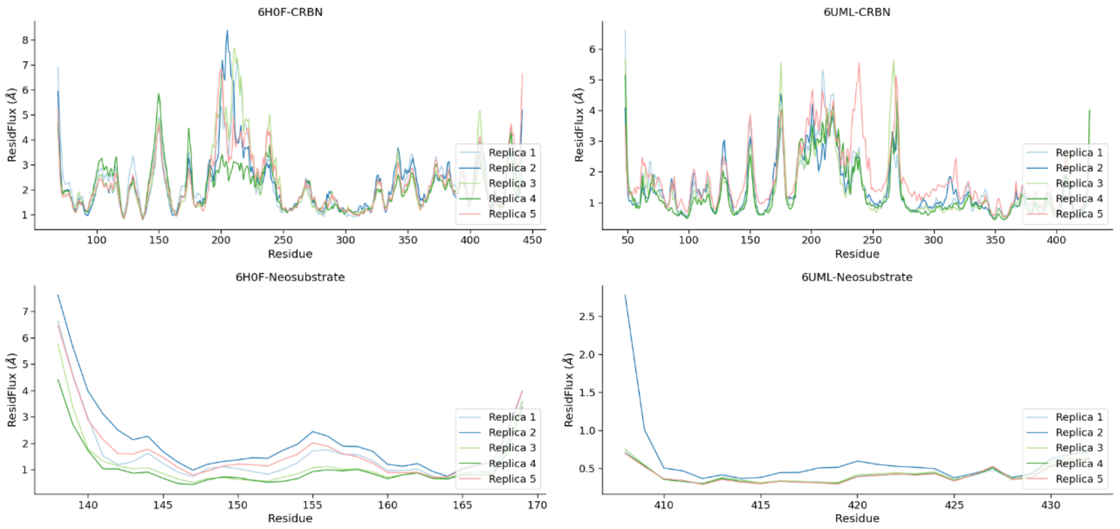

Figure S21 Root-mean square fluctuations (RMSF) of backbone atoms in the complex with pomalidomide for CRBN and neosubstrate. Left panels simulations with SALL4, right panels with IKZF1.

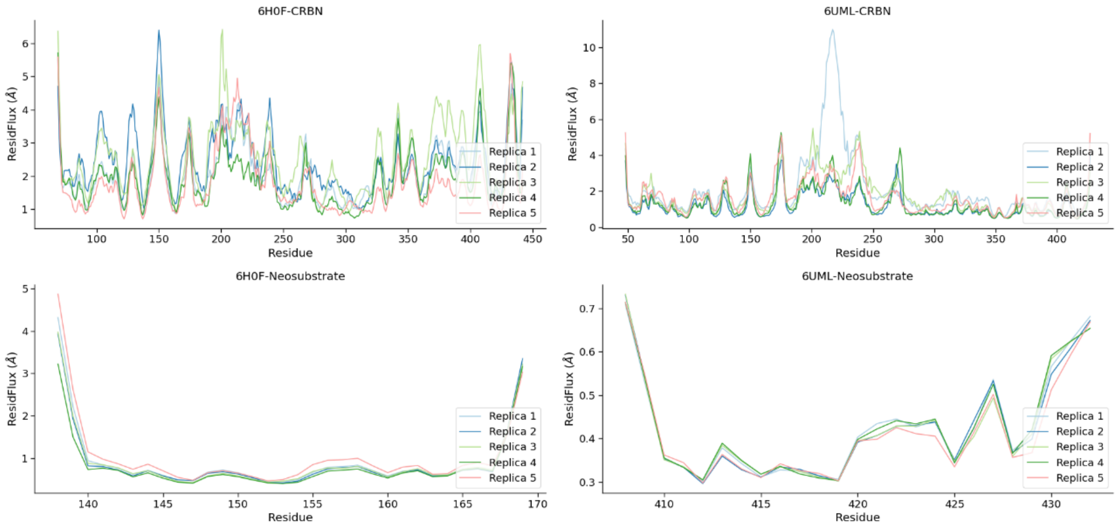

Figure S22 Root-mean square fluctuations (RMSF) of backbone atoms in the complex with thalidomide for CRBN and neosubstrate. Left panels simulations with SALL4, right panels with IKZF1.

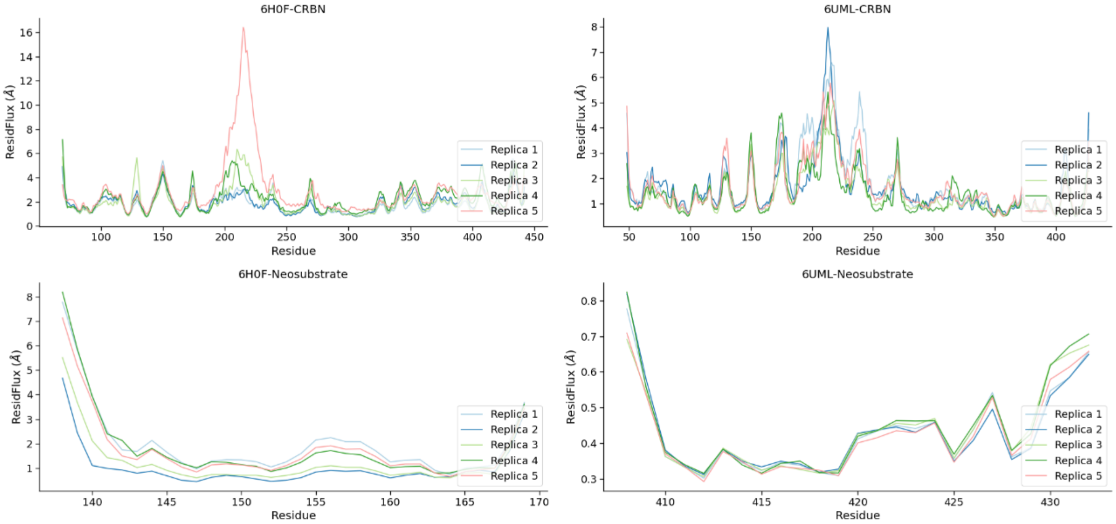

Figure S23 Root-mean square fluctuations (RMSF) of backbone atoms in the complex with 4-hydroxythalidomide for CRBN and neosubstrate. Left panels simulations with SALL4, right panels with IKZF1.

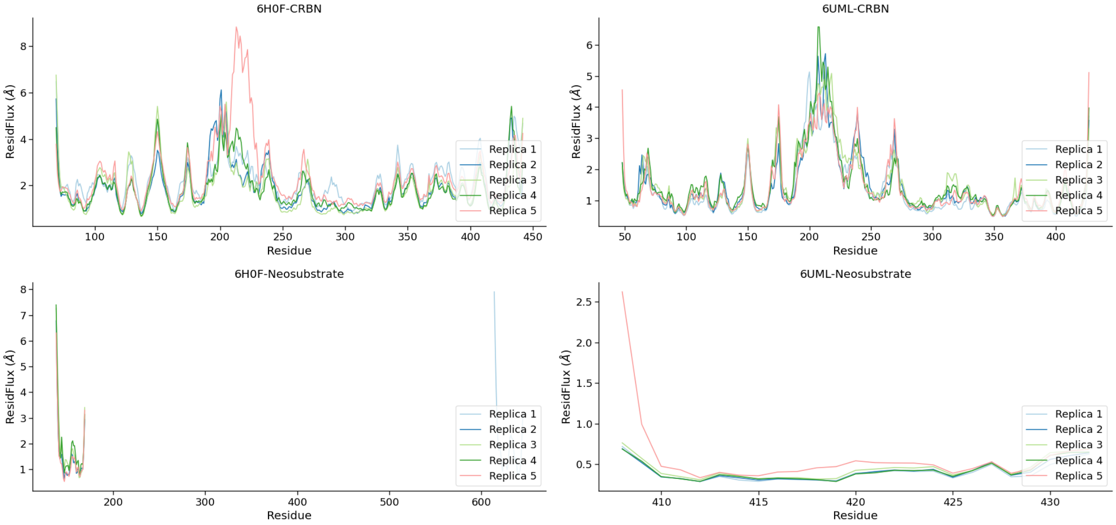

Figure S24. Root-mean square fluctuations (RMSF) of backbone atoms in the complex with 5-hydroxythalidomide for CRBN and neosubstrate. Left panels simulations with SALL4, right panels with IKZF1.

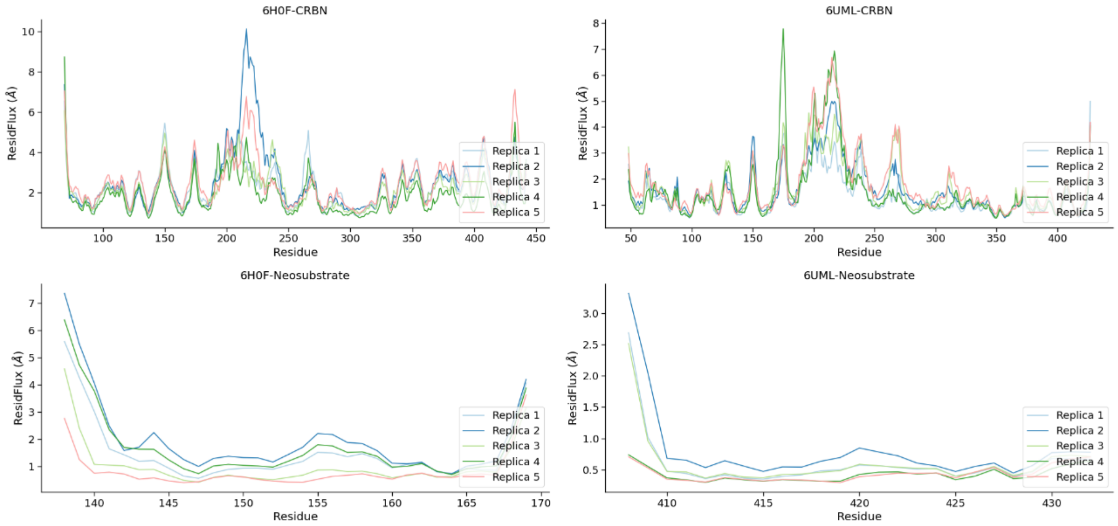

Figure S25 Root-mean square fluctuations (RMSF) of backbone atoms in the complex with lenalidomide for CRBN and neosubstrate. Left panels simulations with SALL4, right panels with IKZF1.
